# A novel plasmid-encoded transposon-derived small RNA reveals the mechanism of sRNA-regulated bacterial persistence

**DOI:** 10.1128/mbio.03814-24

**Published:** 2025-02-25

**Authors:** Shu-Ling Lin, Qi-Chang Nie, Carmen Oi-Kwan Law, Hoa-Quynh Pham, Ho-Fai Chau, Terrence Chi-Kong Lau

**Affiliations:** 1 Department of Biomedical Sciences, College of Biomedicine, City University of Hong Kong, Hong Kong, China; 2 Tung Biomedical Sciences Centre, City University of Hong Kong, Hong Kong, China; 3 Department of Applied Biology and Chemical Technology, The Hong Kong Polytechnic University, Hong Kong, China; The University of Kansas Medical Center, Kansas City, Kansas, USA

**Keywords:** plasmid-encoded sRNA, fosfomycin persistence, fosfomycin transporter YadG, sRNA–transporter interaction

## Abstract

Small regulatory RNAs (sRNAs) in bacteria are crucial for controlling various cellular functions and provide immediate response to the environmental stresses. Antibiotic persistence is a phenomenon that a small subpopulation of bacteria survives under the exposure of a lethal concentration of antibiotics, potentially leading to the development of drug resistance in bacteria. Here, we reported a novel transposon-derived sRNA called stnpA, which can modulate fosfomycin persistence of the bacteria. The stnpA sRNA located in the transposon with its own promoter is highly conserved among the prevalent multidrug resistance (MDR) plasmids in various pathogenic bacteria and expressed in response to the fosfomycin stress. It can directly bind to the ABC transporter, YadG, whereas this protein–RNA interaction modulated the export of fosfomycin and led to the enhancement of bacterial persistence. According to our knowledge, stnpA is the first identified transposon-derived sRNA, which controlled antibiotic persistence of bacteria, and our work demonstrated that nonresistance genes on MDR plasmids such as plasmid-encoded sRNA can provide additional survival advantages to the bacterial host against the antibiotics. In addition, the stnpA sRNA can be potentially utilized as the druggable target for the development of novel therapeutic strategies to overcome bacterial persistence.

**IMPORTANCE:**

This study unveils a groundbreaking discovery in the realm of bacterial antibiotic persistence, highlighting the pivotal role of a newly identified small RNA (sRNA) called stnpA, which is a multidrug resistance plasmid-encoded transposon-derived sRNA that interacts directly with ABC transporter YadG to modulate the efflux of fosfomycin. Our findings elucidate a novel mechanism of small RNA-regulated fosfomycin persistence in bacteria that provides the potential pathway for the emergence of drug resistance in bacteria upon antibiotic treatment. Importantly, this study provides the first example of linking sRNA regulation to antibiotic persistence, presenting stnpA sRNA as a potential therapeutic target. This study underscores the critical role of noncoding RNAs in bacterial adaptation and offers valuable insights for developing new strategies to combat antibiotic persistence.

## INTRODUCTION

Plasmids are the self-replicating extrachromosomal elements that frequently carry and spread genes to provide bacteria with specific traits like resistance, virulence, the capacity to metabolize uncommon substances, and survival in harsh environments (1, 2). Conjugative plasmids, in particular, play a crucial role in the development of pathogenic bacteria as they can be transferred horizontally between and within species (3–5). As vectors of horizontal gene transfer, these plasmids significantly contribute to bacterial adaptation by providing fitness benefits to their hosts (6, 7), and therefore, it was considered one of the major pathways to the emergence of multidrug-resistant bacteria (8, 9). In addition to the dissemination of resistant genes by plasmids, the development of drug-resistant bacteria is also attributed to the controlled recombination activity of transposons (Tn) (10). Among the various transposon families, Tn2, which belongs to the Tn3-family transposon, is the most common variant in antibiotic-resistant plasmids (11, 12). Autonomous members of the Tn3 family contain a core transposition module that consists of the transposase gene (*tnpA*) and inverted repeats (IRs) at the ends of the transposon (13, 14). Full-length Tn3-family *tnpA* genes are unusually long (around 3,000 bp) compared to other transposase genes. Intriguingly, truncated forms of TnpA protein missing their DD(E/D) catalytic domain, which lost the transposition activity, were commonly found on the conjugative plasmids in various *Enterobacteriaceae* such as *Escherichia coli*, *Klebsiella pneumoniae*, *Citrobacter freundii,* and *Enterobacter cloacae* (15–17). Truncated transposons were recently found to have a significant impact on host gene expression by introducing novel regulatory sequences, polyadenylation signals, and additional transcription factor binding sites, as well as in post-transcriptional regulation through RNA editing and translation control (18, 19). Several small RNAs (sRNAs) derived from transposon regions were reported to play regulatory roles in different biological processes (20–23). In bacteria, sRNAs are typically short, noncoding molecules located within the genomes or mobile and accessory elements and regulate the expression of target genes by pairing with RNAs or forming complexes with proteins, usually influencing the stability and translation of mRNAs or modifying the activity of proteins (24–26). They are often expressed under specific conditions or environmental stresses (27) and provide immediate responses of physiological functions such as stress responses, motility, biofilm formation, and metabolic processes to the host (28, 29). In various gram-positive bacteria, sRNAs also control genes related to quorum sensing and virulence factors, underscoring their significance in pathogenesis (30, 31).

Apart from the dissemination of resistant genes and regulation of sRNAs, growing concerns regarding the escalating rates of antibiotic treatment failure and advancements in single-cell analyses have sparked a wave of investigations into antibiotic persistence, a phenomenon that refers to the noninherited tolerance of a subset of bacteria to high levels of antibiotics (32). The size and composition of the persister subpopulation within bacterial communities are predominantly regulated by stress signaling pathways, including the general stress response and SOS response. Consequently, conditions that promote the activation of these signaling pathways, such as bacterial biofilms, hostile host environments, and exposure to sublethal antibiotic concentrations, trigger the formation of persisters (33). However, the comprehensive understanding of the molecular mechanisms governing persister formation, survival, and resuscitation, which led to bacterial persistence, remains unclear. Accumulation of antibiotics represents a key aspect of bacterial tolerance. In gram-negative bacteria, two factors play a crucial role in determining the intracellular level of antibiotic accumulation: membrane permeability and efflux activity (34, 35). Fosfomycin is commonly prescribed for treating acute urinary tract infections, and this antibiotic penetrates the bacterial cytoplasm and hinders peptidoglycan biosynthesis by targeting the MurA enzyme. Initial work showed that fosfomycin could be transported into cells by two main transport systems: the l-alpha-glycerophosphate and the hexose-6-phosphate transporter systems (GlpT and UhpT, respectively) (36). Recent studies also indicated that outer membrane porins OmpF, OprP, and OprO can facilitate the permeation of fosfomycin across the membrane in gram-negative bacteria (37). Currently, it is known that bacteria possess five major superfamilies of membrane transporters, including the ATP-binding cassette superfamily (ABC), major facilitator superfamily (MFS), small multidrug resistance family (SMR), resistance–nodulation–cell division superfamily (RND), and multi-antimicrobial extrusion protein family (MATE) (35). Among them, the ABC superfamily was considered the predominant transporters across all life domains, playing vital roles in various biological processes by transporting a wide range of substances like antibiotics, lipids, and proteins across cell membranes in an ATP-dependent manner (38–40). YadG is the ATP-binding protein of a putative ABC transporter, which belongs to the type III transporter with the predicted functions of antibiotic export and virulence (41). It has been found to expel antibiotics and disinfectants from the cell to the external environment (42, 43).

In this study, we identified and characterized a plasmid-encoded sRNA (stnpA) that modulated fosfomycin persistence of bacteria. This sRNA was derived from the 3' end of the transposase of the Tn3-family transposon encoded on the prevalent multidrug-resistant plasmid pNDM-HN380 and was expressed in response to fosfomycin treatment. Moreover, the stnpA sRNA bound directly to the YadG protein and modulated the accumulation of the fosfomycin in the cells, leading to bacterial persistence against fosfomycin. To our knowledge, the role of sRNAs in bacterial persistence has rarely been reported, and this work is the first to demonstrate the MDR plasmid-encoded sRNA that regulated antibiotic persistence to provide additional survival advantages to the bacteria against antibiotics.

## MATERIALS AND METHODS

### Bacterial strains, plasmids, and growth conditions

A list of all bacterial strains, plasmids, and primers used can be found in Tables S1 and S2. *Escherichia coli* (*E. coli*) strain DH5α was employed for routine cloning and plasmid propagation. *E. coli* BL21(DE3) was utilized for recombinant protein expression. *E. coli* J53 was chosen to explore the functions and mechanisms of action of stnpA, and uropathogenic *Escherichia coli* (UPEC) CFT073 was used to further evaluate the impact of stnpA on virulence and pathogenesis in pathogenic *E. coli*. Mutant strains and mutant pNDM-HN380 were generated through λ red recombineering and were validated using colony PCR and Sanger sequencing. Briefly, deletions were achieved by substituting the target sequence with a kanamycin cassette using the λ red recombinase system, followed by the removal of the kanamycin resistance marker through Flp/FRT excision. Plasmids were constructed by amplifying the target sequences from the genomic DNA of J53 and pNDM-HN380 as templates, and the amplified sequences were then inserted into the desired vectors using either the ClonExpress II One Step Cloning Kit (Vazyme) or by digestion with specified restriction enzymes, followed by ligation using T4 DNA ligase (New England Biolabs).

The strains employed in this study were grown in lysogeny broth (LB) medium or on LB agar plates at 37°C under aerobic conditions, unless specified otherwise. Antibiotics and inducers were added to the liquid and solid media as needed.

### RNA extraction

RNAs used for northern blot and rapid amplification of cDNA ends (RACE) were extracted by TRIzol (Invitrogen). Briefly, 50 mL of bacterial cells from the culture with an OD_600_ of 0.6–0.8 was harvested and then resuspended in 1 mL of TRIzol. The resuspended cells were then incubated at 60°C for 10 minutes, after which the samples were centrifuged, and the supernatant was collected. Subsequently, the supernatant was mixed vigorously with 200 mL of chloroform and allowed to precipitate for 5 minutes. The mixture was then centrifuged at maximum speed for 15 minutes at 4°C, and around 400 µL of the upper aqueous layer was transferred into a fresh microcentrifuge tube, mixed with 1 mL of isopropanol, and placed at −80°C overnight. The RNA precipitate was collected by centrifugation at maximum speed for 15 minutes at 4°C. The pellet was then washed twice with 500 µL of ice-cold 70% ethanol, air-dried, and dissolved in 80 µL of nuclease-free water. To remove any contaminating DNA, TURBO DNase was added to the RNA sample and incubated for 75 minutes at 37°C. The DNase was subsequently removed by repeating the TRIzol–chloroform extraction process. Finally, the total RNA concentrations were determined by NanoDrop One/Onc^C^ UV–Vis spectrophotometry.

For RNA level analysis, including electrophoretic mobility shift assay, RNA pull-down assay, and reverse transcription quantitative polymerase chain reaction, total RNA was isolated using the Qiagen RNeasy Mini kit, with an on-column DNase digestion using the RNase-free DNase Set (Qiagen), performed according to the manufacturer’s protocol. The integrity of the total RNA was assessed through agarose gel electrophoresis, and quantification was performed using a NanoDrop One/Onc^C^ UV–Vis spectrophotometry.

### Northern blot

For the northern blot assay, total RNA (60 µg) was denatured at 70°C for 10 minutes in Gel Loading Buffer II (Ambion) and loaded onto 6% urea-denaturing polyacrylamide gels. RNAs were then transferred to the Hybond-XL membrane (Amersham) and cross-linked under a UV light at 120 mJ/ cm^2^ for 2 minutes. An *in vitro*-transcribed RNA probe targeting stnpA was radioactively labeled with [γ-^32^P]-ATP using T4 polynucleotide kinase (New England Biolabs, NEB) and subsequently purified using a Centri-Spin column-20 (Princeton Separations). Then, the membranes were hybridized with probes at 42°C overnight after prehybridization with UltraHyb buffer (Ambion), followed by washing twice with 20 mL of 0.2 sodium–sodium citrate (SSC) and 0.1% SDS for 10 minutes. The membranes were then exposed to a phosphor screen overnight and imaged using a PhosphorImager (Typhoon TRIO, Amersham Biosciences).

### Rapid amplification of cDNA ends (RACE)

5' and 3' RACE experiments were conducted utilizing the FirstChoice RLM-RACE Kit (Invitrogen) following the manufacturer’s instruction.

For 5' RACE, 10 µg of total RNA extracted by TRIzol was first treated with tobacco alkaline phosphatase (TAP) for 1 hour at 37°C. Subsequently, the TAP-treated RNA was ligated to the 5' RACE adapter by using T4 RNA ligase for 1 hour at 37°C. Following this, 2 µL of the ligated RNA served as a template for cDNA synthesis with M-MLV reverse transcriptase and random hexamer primer, incubating for 1 hour at 42°C. The cDNA was then subjected to nested PCR, with the first round using the 5' RACE gene-specific outer primer (5' RACE outer-R) and the 5' RACE Outer Primer (5' RACE outer-F). For the second round, the 5' RACE gene-specific inner primer (5' RACE inner-R) and the 5' RACE Inner Primer (5' RACE inner-F) were used. The primer sequences are provided in Table S2. The resulting PCR products were cloned into the pGEM-T Easy vector (Promega) and then sequenced.

For 3' RACE, 4 µg of total RNA extracted by TRIzol was first added of a poly(A) tail to the 3' termini of RNA transcript by using *E.coli* poly(A) polymerase (NEB). After transcription–polyadenylation was carried out, these RNAs were reverse-transcribed to cDNA with a 3' RACE adapter and M-MLV Reverse Transcriptase at 42°C for 1 hour. Following this, the resulting RT reaction was used as the template for nested PCR. The first round of PCR was conducted using the 3' RACE gene-specific outer primer (3' RACE outer-F) and 3' RACE Outer Primer (3' RACE outer-R. The products from this first-round PCR were then used as the template for the second-round PCR, which was conducted using the 3' RACE gene-specific inner primer (3' RACE inner-F) and the 3' RACE Inner Primer (3' RACE inner-R). Finally, the PCR fragments were cloned into the pGEM-T Easy vector (Promega) and then sequenced.

### Protein purification

Proteins with an N-terminal His 6-tag were expressed by inserting the corresponding DNA fragments into the pET28a plasmid, which was then transformed into the BL21 (DE3) chemically competent cells. The bacteria were cultured in LB media at 37°C until reaching an OD_600_ of approximately 0.6, followed by induction with a final concentration of 1 mM isopropyl-β-D-thiogalactopyranoside (IPTG). After induction, the cells were allowed to grow for 18 hours at 16°C and then harvested through centrifugation. The cell pellets were then resuspended in lysis buffer (20 mM Tris, 150 mM NaCl, 0.5% (vol/vol) Triton X-100, pH 7.5), supplemented with lysozyme at a final concentration of 100 µL/ mL and lysed by sonication. The resulting lysate was subjected to centrifugation for 20 minutes at 14,000 rpm at 4°C. The supernatant was loaded onto a Ni-NTA column and incubated at 4°C for 1 hour with rotation. Subsequently, the beads were washed twice with wash buffer I (20 mM Tris, 150 mM NaCl, 20 mM imidazole, pH 7.5) and then twice with wash buffer II (20 mM Tris, 500 mM NaCl, 20 mM imidazole, pH 7.5). The bound protein was eluted using an elution buffer (20 mM Tris, 150 mM NaCl, 250 mM imidazole, pH 5.0). The eluted protein solutions were buffer-exchanged and concentrated into storage buffer (10 mM Tris buffer, 150 mM NaCl, pH 7.5) using an Amicon ultra-centrifugal filter (3 kDa). The purity of proteins was analyzed by SDS-PAGE, and the concentration was determined using the Bradford assay method.

### Western blot

The purified protein samples were resolved on a 10% denaturing sodium dodecyl sulphate-polyacrylamide gel electrophoresis (SDS-PAGE) gel and subsequently transferred to a polyvinylidene difluoride (PVDF) membrane via electroblotting. After the transfer, the membrane was blocked in 3% bovine serum albumin (BSA) in TBST (Tris-buffered saline containing 0.1% Tween-20) for 1 hour at room temperature, followed by overnight incubation in 3% BSA in TBST at 4°C with anti-His tag primary antibody (sc-8036, SantaCruz) at 1:1,000 dilution. The incubated membrane was washed three times with TBST before incubation with the HRP-linked secondary anti-mouse IgG antibody (7076S, Cell Signaling Technology) at 1:1,000 dilution in TBST with 3% BSA at room temperature for 1 hour. After washing, the membrane was incubated in a chemiluminescence substrate (Bio-Rad) for 5 minutes, and images were captured by ChemiDoc (Bio-Rad).

### Persistence assay

Overnight bacterial cultures were diluted 1:100 in fresh LB containing appropriate antibiotics and incubated at 37°C with shaking for around 3 hours to the exponential growth phase. For these protein overexpression strains, cultures in the exponential growth phase were split, with one half induced with IPTG (1 mM, 2 hours) and the other half left uninduced as a control. The bacteria were then exposed to fosfomycin at a final concentration of 100 µg/mL at 37°C. Colony-forming unit (CFU) counts were determined by serial dilution and plating on LB agar supplemented with appropriate antibiotics. The percentage of cell viability was calculated by dividing the number of CFU/mL in the culture after exposure to the antibiotic by the number of CFU/mL in the culture before antibiotic addition.

### RNA pull-down assay

Templates of sense and antisense stnpA RNAs with a T7 promoter were obtained by PCR amplification from the pNDM-HN380 plasmid using the primers, including stnpA-*in vitro*-F, stnpA-*in vitro*-R, antisense stnpA-*in vitro*-F, and antisense stnpA-*in vitro*-R. PCR products were purified by the use of the GFX PCR DNA and Gel Band purification kit (GE Healthcare). Purified DNA was transcribed *in vitro* by using the MEGAshortscript T7 Kit (Ambion) incorporating Bio-11-UTP (Ambion) at a final concentration of 0.1 mM. Transcripts were purified by gel purification. The quality of the *in vitro*-transcribed RNAs was verified on 6% TBE-Urea gel by Gel Red staining, and the concentration was quantitated by a NanoDrop One/Onc^C^ UV–Vis. Purified biotinylated RNAs (3 pmol) were incubated with 50 µL of equilibrated streptavidin magnetic beads in 950 µL of binding buffer (100 mM KCl, 5 mM MgCl_2_, 20 mM Tris-HCl pH 7.4, 0.5% NP40) for 1 hour at 4°C with rotation. After incubation, the beads were washed with binding buffer and then resuspended in 50 µL of binding buffer.

Bacterial lysates were prepared by sonicating the cells in a binding buffer supplemented with protease inhibitors. The lysate was centrifuged at 14,000 rpm for 20 minutes at 4°C; filtered through 0.22-µm filter paper; then added to the above-prepared beads-biotinylated RNA complex supplemented with 1 × protease inhibitor, 2 µL of RNasin Ribonuclease Inhibitor, 30 µL of yeast tRNA (25 mg/mL), and binding buffer in a final volume of 1 mL; and incubated for 2 hours at 4°C with intermittent pipetting up and down every 10 minutes. Then, beads were washed four times with washing buffer (100 mM NaCl, 2 mM EDTA pH 8.0, 20 mM Tris-HCl pH 8.0, 1% Triton X-100). The bound proteins were eluted by boiling the beads in 50 µL of 50 mM Tris buffer (pH 8.0) containing 8M urea for 10 minutes and subjected to silver staining or identified by mass spectrometry.

### Reverse transcription–quantitative polymerase chain reaction (RT-qPCR)

An amount of 1 µg of RNA was transcribed onto cDNA utilizing a PrimeScript First-Strand cDNA Synthesis Kit (Takara). The amplified cDNA was diluted 10 times in nuclease-free water. Real-time PCR analysis was conducted using the SYBR Green PCR master mix (Applied Biosystems) on a QuantStudio 5 Real-Time PCR system (Thermo Fisher Scientific). A minimum of three biological replicates were analyzed in technical triplicate in each experiment, with the gapA gene serving as the reference gene for relative quantification. Relative changes in gene expression were determined by the 2^−ΔΔCt^ method.

### Promoter study

The region 290 bp upstream of the stnpA transcription start site was amplified using primers PstnpA-F and PstnpA-R and cloned into the promoterless bioluminescence reporter plasmid pQH5 to construct pQH5-*stnpA*. Subsequently, point mutations were introduced into the predicted −10 and −35 core promoter elements of the stnpA promoter using PCR-directed mutagenesis with primers SMu-1-R to SMu-6-R. The plasmids were transformed into CFT073 for bioluminescence measurement. Overnight cultures were diluted 100-fold in fresh LB broth containing chloramphenicol at 37°C with shaking for around 3 hours until an OD_600_ of 0.6 was reached. Cellular bioluminescence and absorbance at 600 nm (OD_600_) were measured using a microplate reader (BioTek, Synergy H1). Promoter activity was presented in bioluminescence readings normalized to OD_600_.

### Bacterial three-hybrid (B3H) assay

B3H assay was conducted to detect the RNA–protein interactions *in vivo*. The reporter strain *E. coli* strain HP16 (Kan^R^) contains a *lacZ* reporter gene, whose expression depends on the specific RNA–protein interaction being studied, allowing the B3H assay to serve as a reporter system. This reporter strain was transformed with three plasmids: one (p35u4, Cam^R^) constitutive expressing a fusion protein comprising the DNA-binding protein λCI and the RNA-binding coat protein from bacteriophage MS2, which acts as an RNA–DNA adapter in the B3H system; another (pBRα-*yadG*, Amp^R^) expressing the protein of interest (YadG) linked to the N-terminal domain of the *E. coli* RNAP α subunit under the control of IPTG; and a third one (pCH1-*stnpA*, Str^R^) expressing a hybrid RNA containing an MS2 hairpin upstream of the sRNA of interest (stnpA) under the control of arabinose, with antisense stnpA construct (pCH1-*antisense stnpA*) as the negative control. For each transformation, three individual colonies were selected and inoculated into 1 mL of LB broth containing ampicillin (100 µg/ mL), chloramphenicol (34 µg/ mL), streptomycin (100 µg/ mL), kanamycin (50 µg/ mL), and 0.2% arabinose. The cultures were incubated at 37°C with shaking at 220 rpm for overnight. The overnight cultures were diluted 1:100 into 4 mL of the LB medium as described above, along with an additional 50 µM of IPTG, and allowed to grow until reaching the mid-log phase (OD_600_ of approximately 0.6).

For the liquid-based assay, β-gal activity was measured using the β-Gal assay kit (Thermo Fisher) according to the manufacturer’s instruction. Briefly, 1 mL of the mid-log cells was washed once with 1 × PBS and subsequently resuspended in 100 µL of 1 × lysis buffer. The lysis process was done by repeatedly freezing the samples on dry ice and thawing at 37°C twice. Following centrifugation at maximum speed for 5 minutes, 10 µL of the supernatant was transferred to a fresh microcentrifuge tube and mixed with 20 µL of distilled, deionized water. Then, 70 µL of ONPG and 200 µL of 1 × Cleavage Buffer with β-mercaptoethanol were added to the mixture, followed by incubation at 37°C for 30 minutes. Once the sample developed a faint yellow color, the reaction was terminated by adding 500 µL of stop buffer. Subsequently, 150 µL of the reaction mixture from each sample was transferred to a 96-well plate for measurement of OD_420_ using a microplate reader (BioTek, Synergy H1). β-gal activity was calculated according to the following formula: Miller units = $\left(1000\times OD_{420}\right)/\left(T\times V\times OD_{600}\right)$, where *T* = time of reaction (minutes); *V* = volume of culture used in assay (mL); OD_600_ = cell density before lysis.

For the plate-based assay, 5 µL of the mid-log cells at 1:1,000, 1:10,000, and 1:100,000 dilution was plated on an LB agar plate supplemented with X-gal (40 µg/mL) and TPEG (200 µM) along with IPTG, arabinose, and antibiotics as mentioned above and incubated at 37°C overnight, respectively. After this incubation period, plates were kept at 4°C for 1–2 days for color development before being photographed. The assessment of β-gal activity was based on the relative color intensity of bacterial patches on the X-gal indicator plate, where a deeper blue color indicated higher β-gal activity and thus a more robust RNA–protein interaction.

### Electrophoretic mobility shift assay (EMSA)

stnpA was *in vitro* transcribed from PCR-amplified DNA utilizing a MEGAshortscript T7 Kit (ambion), while the DNA template was ampliﬁed from pNDM-HN380 plasmid using the primers stnpA-*in vitro*-F and stnpA with SP6-*in vitro*-R. These primers were designed such that the amplicons had the T7 promoter sequence at the 5' end and the reverse complementary sequence of the SP6 promoter at the 3' end. The transcribed RNA was gel-puriﬁed, and the concentration was determined by NanoDrop One/Onc^C^ UV–Vis. About 40 pmol of the *in vitro-*transcribed stnpA was denatured at 90°C for 3 minutes and followed by annealed with 80 pmol of 5' fluorescein (FAM)-labeled SP6 DNA primer (SP6-FAM, Invitrogen) at 25°C for 15 minutes.

For EMSA assays, varying concentrations of purified recombinant protein YadG were, respectively, incubated with 3 pmol FAM-labeled stnpA in binding buffer (100 mM KCl, 5 mM MgCl_2_, 20 mM Tris-HCl pH 7.4, 0.5% NP40) supplemented with tRNA (final concentration at 1 mg/mL) and BSA (final concentration at 1 mg/mL) as well as an RNase inhibitor at a final volume of 40 µL for 30 minutes at room temperature. Following the incubation period, 3 uL of 80% glycerol was introduced into the mixture, and the samples were immediately loaded on a 6% nondenaturing polyacrylamide gel. The image was analyzed using a Typhoon 8600 imager (GE Healthcare). The fraction of bound RNA was measured by densitometry using ImageJ and plotted using nonlinear regression based on one-site specific binding in GraphPad Prism 9.0.2.

### Intracellular fosfomycin accumulation quantification

The assay to determine fosfomycin accumulation in bacterial cells was performed as described in the previous publication (44), with some modifications. Briefly, 1 mL of overnight cultures were inoculated into 100 mL of fresh LB containing appropriate antibiotics and cultivated until reaching the mid-log phase in a 37°C shaking incubator. About 20 mL of bacterial cultures was pelleted by centrifugation and resuspended in 1  mL of the LB medium. Afterward, fosfomycin was then added to the bacterial suspension to a final concentration of 2 mg/mL. Following incubation at 37°C for 30 minutes, extracellular fosfomycin was removed by three washes with PBS. Cells were then resuspended in 1 mL of ultrapure water, and 10 uL of the suspension from each sample was taken to perform serial dilution plating for CFU determination.

To release the intracellular fosfomycin, cells were lysed by sonication. The cell debris was then removed via centrifugation followed by filtration through a 0.22-µm filter. Further deproteinization was achieved using an Amicon Ultra centrifugal filter (3 k). The quantification of fosfomycin levels in the supernatant was conducted through liquid chromatography-tandem mass spectrometry (LC-MS/MS), employing an Agilent 1290 UHPLC system connected to an Agilent 6460 Triple–Quadrupole Mass Spectrometer (Santa Clara, CA). Chromatographic separation was achieved with an Acquity UPLC BEH HILIC column (2.1 × 75 mm, 1.7 µm) (Waters Corporation, Milford, USA) with a guard column, Acquity UPLC BEH HILIC VanGuard Pre-Column (2.1 × 5 mm, 1.7 µm) (Waters Corporation, Milford, USA) at 40°C with a run time of 8 minutes. The mobile phase consisted of 2  mM ammonium acetate (pH 4.8) (A) and acetonitrile (B) at a flow rate of 0.3 mL/min in the gradient mode. The interface was an electrospray ionization (ESI) source, and the negative ion mode was applied. Multiple reaction monitoring (MRM) at m/z 137/79 (quantifier) and m/z 137/63.2 (qualifier) was employed for fosfomycin detection. Quantitation of fosfomycin concentration was accomplished based on the established calibration curve and the peak area of the analyte, while intracellular accumulation was calculated using the formula: *A*=(C×V)/CFUs, where *C* represents the final fosfomycin concentration (μg/ mL), *V* is the final volume of 1 mL, CFUs is the number of colony-forming units, and *A* is accumulation in μg/10^6^ CFUs.

## RESULTS

### Identification and characterization of the ESBL plasmid-encoded small RNA, stnpA

**Fig 1:**
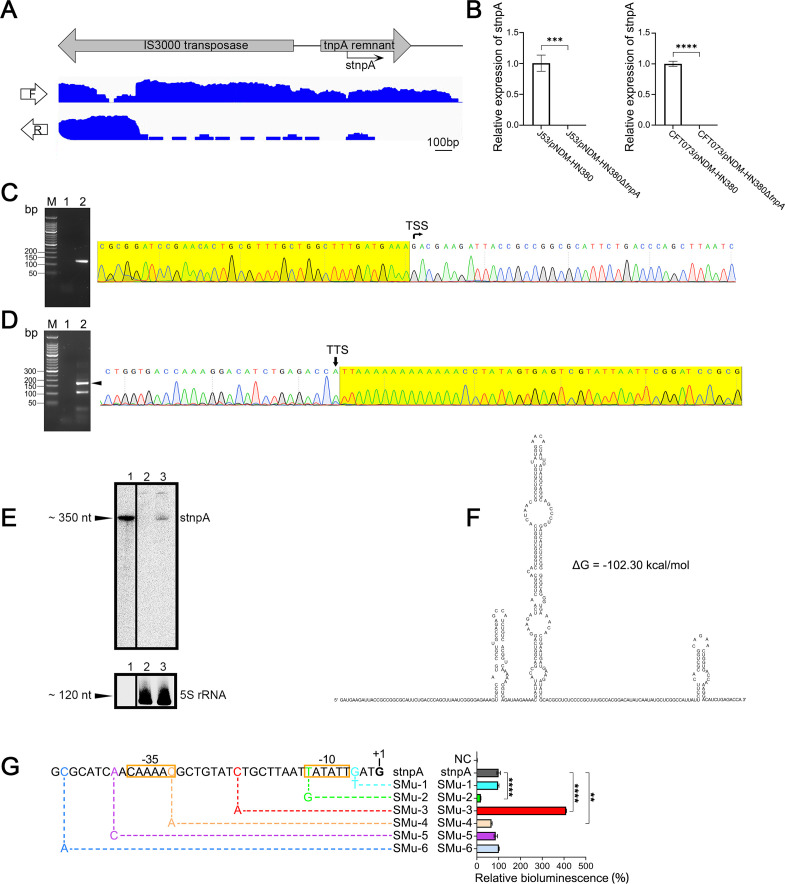
Identification of a Tn2-tnpA-derived sRNA (stnpA). (**A**) IGV tracks for stnpA from RNA-seq data in J53/pNDM-HN380. The upper panel indicates the location of genes on pNDM-HN380, with arrows denoting the direction of transcription. The bottom panel shows reads mapping to the forward (**F**) and reverse (**R**) strands separately. (**B**) Verification of stnpA expression in J53 and CFT073 strains containing pNDM-HN380 compared with its Δ*tnpA* mutant by qPCR, *N* = 3. Bars represent the mean of three biological repeats, and error bars indicate standard deviation (SD) from the mean. ***P < 0.001; ****P < 0.0001 (two-tailed paired *t test*). (**C**) 5' RACE reveals the transcriptional start site (TSS) of stnpA. The left panel shows the nest-PCR products of 5' RACE. Lane M, 50 bp DNA Ladder; lane 1, first-round PCR product; lane 2, second-round PCR product. The right panel shows the sequencing result of the second-round PCR product, where the 5' RACE adapter is shaded in yellow. The position of the TSS is indicated by a black arrow. (**D**) Transcription termination site (TTS) mapping of stnpA by 3' RACE. Left panel: Nested-PCR products of 3′ RACE. Lane M, 50 bp DNA Ladder; lane 1, first-round PCR product; lane 2, second-round PCR product. The most abundant isoform is indicated by a black triangle. The presence of additional bands may be due to the existence of alternative polyadenylation sites. Right panel: sequencing alignment of the most abundant isoform, where the 3′ RACE adapter is shaded in yellow and the position of the TSS is indicated by a black arrow. (**E**) Northern blot analysis of stnpA. Lane 1: *In vitro* transcribed 342 nt RNA; lane 2: RNA from J53/pNDM-HN380Δ*tnpA* cells; lane 3: RNA from J53/pNDMHN380 cells. 5S rRNA was used as a loading control. (**F**) Secondary structure of stnpA was predicted by the web tool Mfold. –ΔG value indicates the stability of the secondary structure. (**G**) Promoter analysis of stnpA. The promoter activity was assessed using a bioluminescent reporter system in which the stnpA promoter region was fused with the promoterless *luxCDABE* operon on a low-copy-number plasmid (pQH5). Mutations (SMu-1 to SMu-6) were introduced to analyze the −10/–35 promoter elements. The promoterless *lux* vector pQH5 served as a negative control (NC). The −10 and −35 elements of the stnpA promoter are indicated.

In our previous RNA-seq analysis of bacteria carrying different drug resistance plasmids, we have identified numerous small RNA (sRNAs) encoded on the prevalent ESBL plasmids (45, 46). Among them, intriguingly, we identified a novel and widely spread sRNA called stnpA located on the remnant gene of transposase *tnpA* upstream to the IS3000 transposase (Fig. 1A). To validate the presence of this putative sRNA, the expression level was measured by the quantitative polymerase chain reaction (qPCR) using specific primers of *stnpA*. The null *tnpA* pNDM-HN380 plasmid (Δ*tnpA*) was used as the control. As shown in Fig. 1B, the expression of the stnpA sRNA in both *E. coli* strains J53 and CFT073 carrying pNDM-HN380Δ*tnpA* plasmids was abolished compared with the wild-type plasmids.

To identify the transcriptional start site (TSS) and transcription termination site (TTS) of stnpA sRNA, 5' and 3' RACE analyses were performed. The results indicated that the size of stnpA is 342 nt (Fig. 1C and D). To further confirm both the existence and size of stnpA, we performed northern blot analysis using a radioactively labeled probe targeting stnpA. As demonstrated in Fig. 1E, a distinct band of approximately 350 nucleotides (nts) was detected in the J53/pNDM-HN380, but not in J53/pNDM-HN380Δ*tnpA* strains. These findings collectively corroborated the expression of stnpA with the length of 342 nucleotides, which encoded within the tnpA region of the pNDM-HN380 plasmid. The predicted secondary structure of stnpA, obtained using MFold (47), is shown in Fig. 1F.

Through sequence analysis and experimental validation, we identified a potential σ^70^-dependent promoter upstream of the *stnpA* gene, which is 5′-TATATT-3′ (−10) and 5′-CAAAAC-3′ (−35). Moreover, various mutations (SMu-1 to SMu-6) at different positions within the promoter region were introduced to assess their effects to the promoter activity using a bioluminescent reporter system. Notably, SMu-2 and SMu-4 mutants which correspond to single-nucleotide mutation on the −10 and −35 motifs, respectively, showed significant reduction in bioluminescence signals, indicating the regulatory control of this promoter to the expression of *stnpA* gene (Fig. 1G).

### stnpA is a small RNA commonly found in prevalent multidrug-resistant plasmids

**Fig 2:**
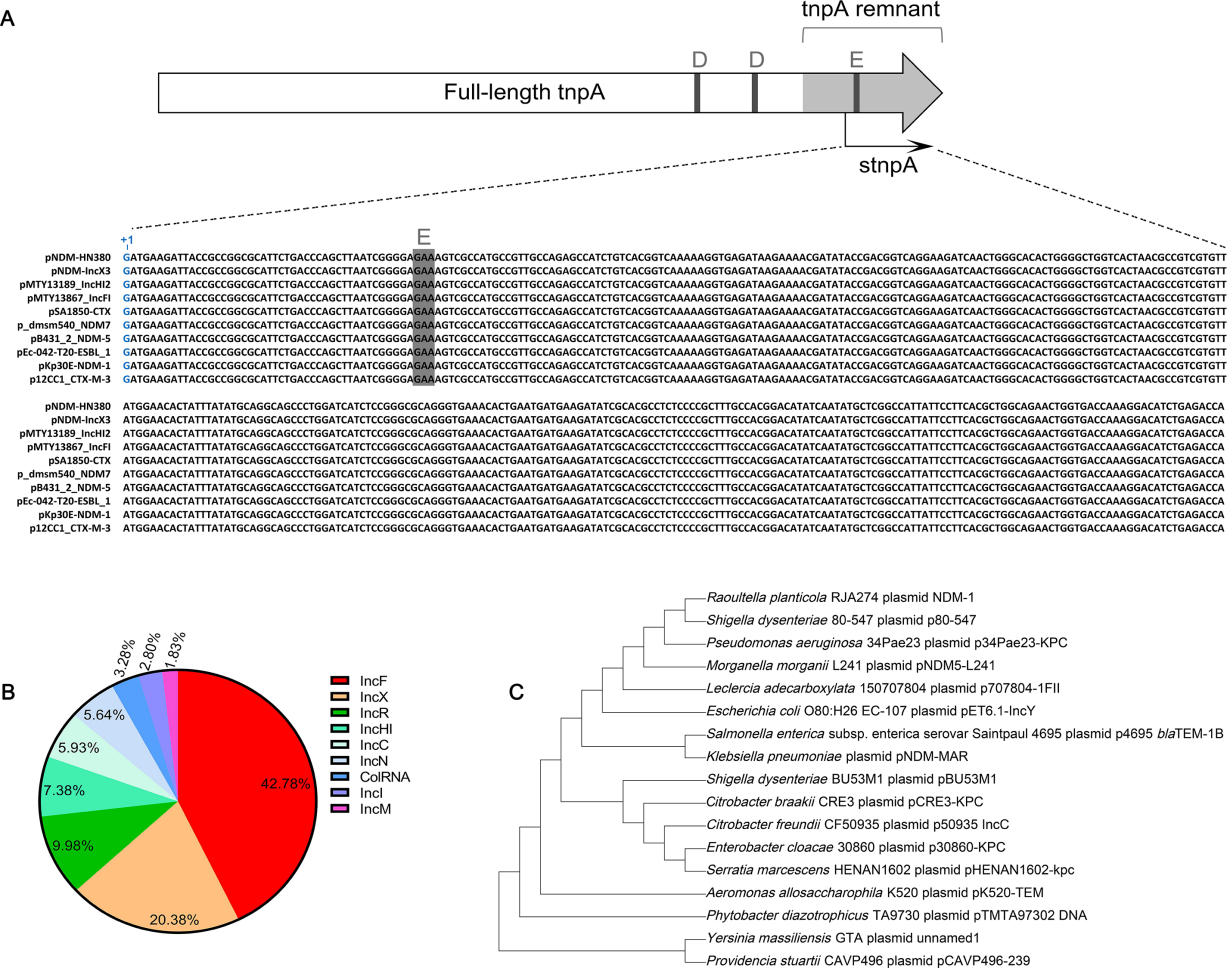
stnpA is a conjugative plasmid-carried sRNA derived from the *tnpA* gene of the Tn2 transposon present in various pathogenic bacteria. (**A**) Sequence alignment of stnpA in partial of the conjugative plasmids that share 100% sequence identity. The vertical bars represent the residues of the DDE catalytic motif. The transcription start site of stnpA is located downstream of the second D residue. (**B**) Pie chart shows the proportion of incompatibility groups of plasmids carrying stnpA. (**C**) stnpA is conserved among pathogenic bacteria. The phylogenetic tree of stnpA with representative microbes was generated based on ClustalW2 multiple sequence alignment. Phylogenetic analysis was performed using the neighbor-joining method and Kimura-2-parameter with 1,000 bootstrap replicates in MEGA X.

Identification of the transcription cassette of stnpA in the pNDM-HN380 plasmid indicated that this sRNA is transcribed in the middle of the partial Tn2 *tnpA* gene, the truncated gene that cannot be expressed in the cell. Moreover, the BLAST analysis against nucleotide collection databases (48) indicated that the *stnpA* gene is highly conserved among other multidrug-resistant (MDR) plasmids (Fig. 2A). Moreover, as shown in Fig. 2B, the *stnpA* gene is mainly harbored on the prevalent MDR plasmids including IncF (42.78%) and IncX (20.38%) groups, which are often found in pathogenic strains and related to the dissemination of antimicrobial resistance and virulence genes (49, 50). Indeed, Inc plasmids have a narrow spectrum of hosts, which are predominantly *Enterobacteriaceae*, and confer fitness benefits to their host (51–53). To analyze the conservation of *stnpA*, a phylogenetic tree was constructed, and the result showed that *stnpA* is conserved among diverse conjugative plasmids in a variety of pathogens such as *Providencia stuartii*, *Pseudomonas aeruginosa*, and *Shigella dysenteriae* (Fig. 2C).

### Fosfomycin stress-induced expression of stnpA sRNA

**Fig 3:**
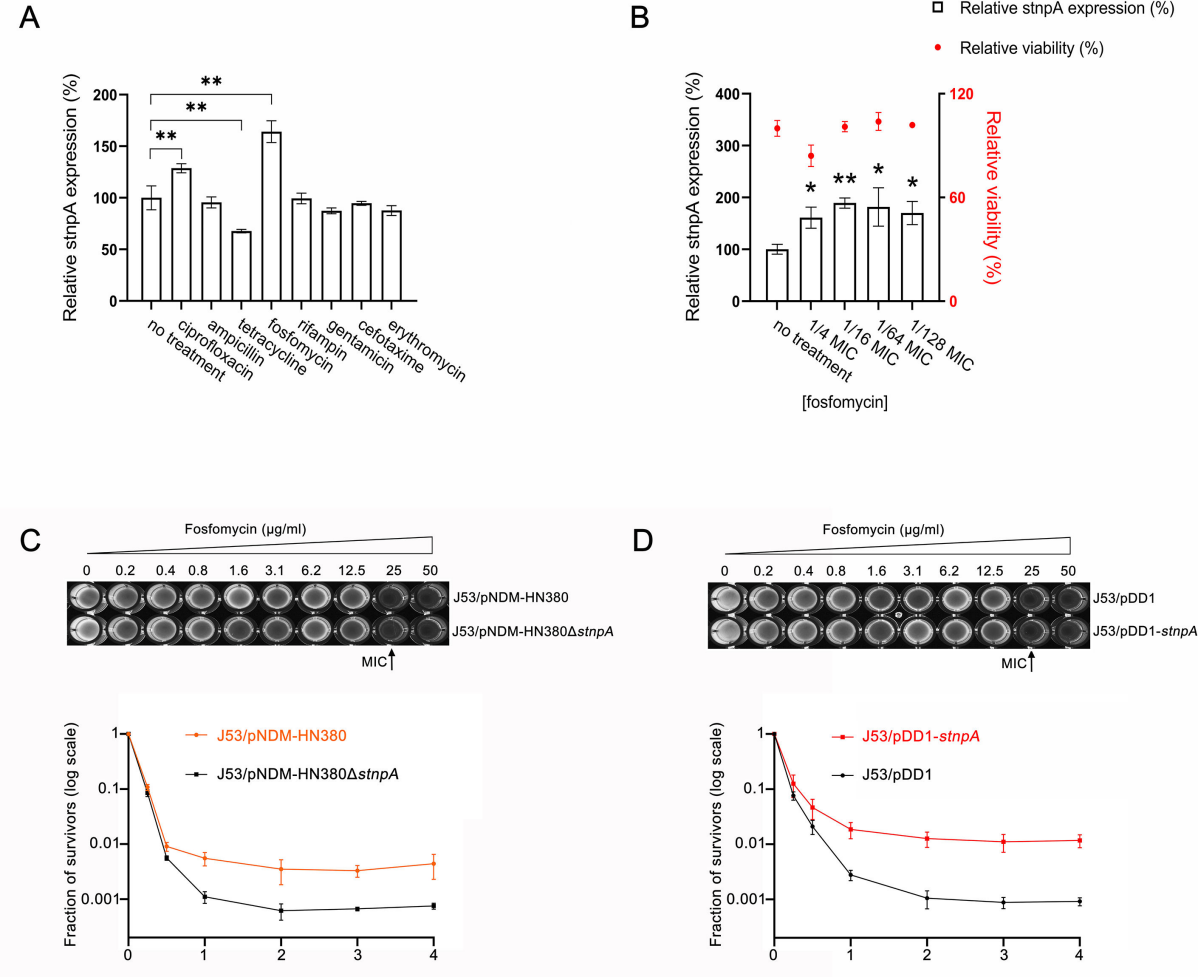
stnpA is involved in response to fosfomycin stress. (**A**) Effect of sub-MIC antibiotics on the expression of stnpA. Log-phase CFT073/pNDM-HN380 cells were treated with and without 1/4 MIC of different antibiotics (ciprofloxacin, ampicillin, tetracycline, fosfomycin, rifampin, gentamicin, cefotaxime, and erythromycin) for 30 minutes. The expression level of stnpA was determined by qPCR. All measurements were conducted in biological replicates. Data are presented as mean ± SD. Statistical significance of differences between treatment and control (no treatment) groups was assessed by the two-tailed paired *t test* (**P ≤ 0.01). (**B**) Quantification of the relative stnpA expression levels in log-phase CFT073/pNDM-HN380 cells after treatment with sub-MICs (1/4, 1/16, 1/64, and 1/128 MIC) of fosfomycin by qPCR analysis. Meanwhile, the relative viability of CFT073/pNDM-HN380 cells upon exposure to sub-MICs of fosfomycin was determined by using the colony plate counting method. All data represent mean ± SD of three independent experiments. **P < 0.01; *P < 0.05 (two-tailed paired *t test*). (**C**) Influence of endogenously expressed stnpA on bacterial survival under fosfomycin stress. Upper panel: representative diagram of MIC determination for J53/pNDM-HN380 and J53/pNDM-HN380∆*stnpA* against fosfomycin. The final fosfomycin concentration ranges from 0 to 50 µg/mL. The wells of a 96-well plate were loaded with 100 µL of diluted exponentially growing cultures (OD_600_ of 0.005), along with a series of twofold dilutions of fosfomycin. The plate was incubated at 37°C for 18 hours. All tests were conducted in triplicate. The MIC value was defined as the lowest fosfomycin concentration that inhibits bacterial growth. Bottom panel: time-kill curve of J53/pNDM-HN380 and pNDM-HN380Δ*stnpA* in the presence of fosfomycin. Bacterial cells in the log phase were treated with 100 µg/mL of fosfomycin at 37°C and sampled over time. Survivors were quantified by depositing serial tenfold dilutions onto LB agar plates supplemented with 100 µg/mL ampicillin, followed by overnight incubation at 37°C. The fraction of survivors was determined by the ratio of the number of surviving bacteria after fosfomycin treatment to the number of viable bacteria before treatment. (**D**) Influence of overexpressed stnpA on bacterial survival under fosfomycin stress. Upper panel: representative diagram of MIC determination for J53/pDD1 and J53/pDD1-*stnpA* against fosfomycin. Bottom panel: time-kill curve of J53/pDD1 and J53/pDD1-*stnpA* in the presence of fosfomycin (100 µg/mL).

Previous studies have indicated that sub-inhibitory concentrations (sub-MICs) of antibiotics reflect the conditions that bacteria encounter in the natural environment, which can induce changes in the gene expression to drive the evolution of antibiotic resistance and tolerance (54–56). To investigate whether sub-MICs of antibiotics affect the expression level of stnpA, the *E. coli* CFT073 strain carrying pNDM-HN380 plasmid (CFT073/pNDM-HN380) was first grown to the log phase and treated with sublethal concentrations (1/4 MIC) of various antibiotics including ciprofloxacin, ampicillin, tetracycline, fosfomycin, rifampin, gentamicin, cefotaxime, and erythromycin for 30 minutes. The expression level of stnpA in each sample was then determined by qPCR. As shown in Fig. 3A, only the sublethal concentration of ciprofloxacin and fosfomycin enhanced the expression of stnpA. On the other hand, intriguingly, treatment of tetracycline alleviated the amount of stnpA transcripts. In addition, decreasing concentrations of fosfomycin (1/4, 1/16, 1/64, and 1/128 MICs were equivalent to 4, 1, 0.25, and 0.125 µg/mL, respectively) showed that the stnpA expression was sensitive to as low as 1/128 MIC (Fig. 3B). These results suggested that the gene expression of MDR plasmid in the bacteria was antibiotic-specific (57, 58). Moreover, significant upregulation of stnpA in response to a wide range of sub-MICs of fosfomycin suggested the potential function of stnpA to protect the bacteria against fosfomycin treatment.

To investigate the role of stnpA in response to fosfomycin stress, the bacteria carrying the wild-type plasmid of pNDM-HN380 and null mutant of *stnpA* (pNDM-HN380ΔstnpA) were employed. The minimum inhibitory concentration (MIC) was measured and compared among different strains to investigate the effect of stnpA on the fosfomycin resistance of the bacteria. As shown in the upper panel of Fig. 3C, the expression of stnpA did not change the MIC of fosfomycin. The role of stnpA in fosfomycin persistence was investigated through time-kill assays. Analysis of the time-kill curves revealed that J53/pNDM-HN380 (carrying stnpA) had a similar MDK_90_ (minimum duration of killing 90% of the initial population) to its control strain J53/pNDM-HN380Δ*stnpA* when exposed to a high concentration of fosfomycin (100 µg/mL). Despite the similarity in the time required to kill a large fraction of the bacterial population, the subpopulation of persister cells carrying stnpA (J53/pNDM-HN380) survived better under the fosfomycin treatment compared to the control strain J53/pNDM-HN380Δ*stnpA* (Fig. 3C, bottom panel), highlighting the significant role of stnpA in fosfomycin persistence of bacteria. To further validate the relationship between stnpA and fosfomycin persistence, we compared the bacteria overexpressing stnpA (J53/pDD1-*stnpA*) with the empty vector control (J53/pDD1) and found that the presence of stnpA did not affect the MIC but dramatically enhanced the number of persisters under the fosfomycin treatment (Fig. 3D). In other words, the presence of the MDR plasmid does not only disseminate the resistant genes but also provides additional survival advantages to the bacterial host against the antibiotics via the promotion of fosfomycin-tolerant persisters, as exemplified here.

### Interaction between stnpA sRNA and the putative ABC transporter YadG

**Fig 4:**
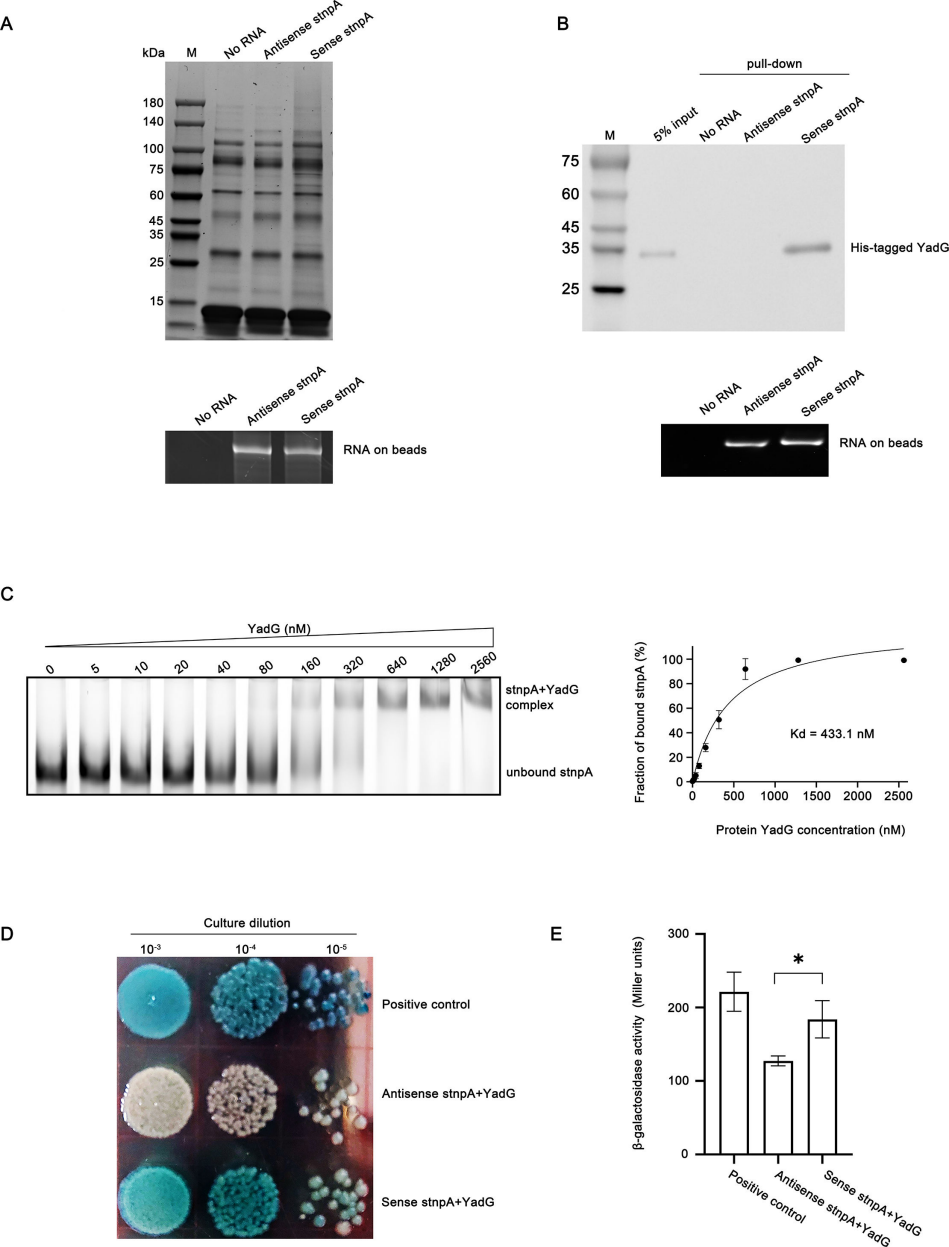
stnpA binds directly with YadG. (**A**) *In vitro* RNA pull-down assay. CFT073 cell lysates were incubated with biotinylated RNA oligos shown in the bottom panel. The RNA–protein complexes captured by streptavidin beads were subjected to silver staining after separation on SDS-PAGE gel. The bottom panel shows the immobilization of biotinylated sense and antisense stnpA on equal volumes of streptavidin beads. About 1 µL of the beads-biotinylated RNA complex was mixed with 4 µL of nuclease-free water and 5 µL of Gel Loading Buffer II (Thermo Fisher) and subjected to an incubation at 65°C for 5 minutes, and subsequently loaded onto an 8% urea–PAGE gel for electrophoresis. No RNA control: streptavidin beads without binding RNAs incubated with binding buffer. (**B**) Western blot to determine the specific interaction of stnpA with YadG protein. Biotin-labeled stnpA and antisense stnpA (negative control) were used for *in vitro* RNA pull-down reactions with purified His-tagged recombinant YadG. Streptavidin beads only (no RNA) served as another negative control. The input represents 5% of the purified recombinant YadG used for *in vitro* RNA pull-down reactions. Western blot was performed using an anti-His tag antibody. The immobilized biotinylated RNAs used for Western blot were analysed using an 8% urea–PAGE gel (bottom panel). (**C**) EMSA of FAM-labeled stnpA (3 pmol) in the presence of increasing concentrations of YadG protein, as indicated. Bands corresponding to unbound stnpA and the stnpA-YadG complex are denoted in the left panel. Quantitative analysis of three repeats of the EMSA is shown in the right panel. The fraction of bound stnpA was plotted against the YadG concentration in nM to calculate the equilibrium dissociation constant, Kd. Data are presented as mean ± SD. (**D**) Qualitative plate-based B3H assay detects the interaction between YadG and stnpA. Positive control: reporter strain HP16 expressing ChiX and Hfq; antisense stnpA +YadG: HP16-expressing antisense stnpA and YadG served as the negative control. Blue indicates RNA–protein interaction, and white indicates no interaction. A darker blue color represents a stronger RNA–protein interaction. (**E**) Quantitative liquid-based B3H assay detects the interaction between YadG and stnpA. Data are presented as means ± SD of three replicates. Asterisks indicate significant differences between sample pairs specified by brackets: *P < 0.05 (two-tailed Student’s *t*-test).

It is known that small RNAs exert many of their biological functions by directly interacting with the proteins (24, 59, 60). In order to investigate the mechanism of stnpA in regulating fosfomycin persistence, we performed RNA pull-down assay, followed by mass spectrometric analysis. The biotinylated stnpA RNA immobilized on streptavidin beads was used to pull down the associated proteins and analyzed on SDS-PAGE using silver staining (Fig. 4A). An equal amount of the biotinylated antisense stnpA RNA (same size but reverse and complementary) was used as controls. As shown in Fig. 4A, the biotinylated stnpA RNA can pull down several specific proteins compared with the controls (beads and antisense stnpA). The bands were cut from the gel, purified, and subjected for mass spectrometric analysis (Table S3). Among the top candidates of interacting proteins, identified based on their relative peptide coverage from mass spectrometry, a putative ABC transporter ATP-binding protein, YadG, was selected for further studies due to its relatively high sequence coverage. To confirm the RNA–protein interaction between stnpA and YadG, the biotinylated stnpA RNA was used to perform the RNA immunoprecipitation in bacteria carrying His-YadG protein (Fig. S1) and detected with anti-His antibody in Western blot analysis. The biotinylated antisense stnpA was used as the control. As shown in Fig. 4B, only stnpA RNA can pull down YadG. To further characterize the interaction, electrophoretic mobility shift assays (EMSA) was performed, and the dissociation constant was determined (Fig. 4C). These results indicated that the stnpA RNA recognized the YadG protein with high affinity and specificity.

In addition to the *in vitro* study of stnpA–YadG interaction, we also performed the bacterial three-hybrid (B3H) assay to detect their interaction inside the cells. The B3H assay is the *in vivo* system that reports RNA–protein interactions inside the living *E. coli* cells through the transcription of a reporter gene (61), and the intensity of the color (blue here) represents the strength of the RNA–protein interaction. As shown in Fig. 4D, stnpA RNA interacted with YadG protein in the cells, while no binding was observed in the control (antisense stnpA RNA and YadG protein). The *E. coli* strain expressing ChiX and Hfq was used as the positive control. Indeed, a quantitative liquid-based B3H assay was also performed, as shown in Fig. 4E, and a significantly higher level of β-gal activity was detected in the cells expressing YadG and stnpA compared with the control. Noteworthily, the qualitative detection on the plates appears to offer better sensitivity than liquid β-gal assays (61). Overall, both the *in vitro* and *in vivo* binding experiments demonstrated that stnpA RNA interacted with YadG protein with high affinity and specificity.

### stnpA sRNA enhanced fosfomycin persistence in a YadG-dependent manner

**Fig 5:**
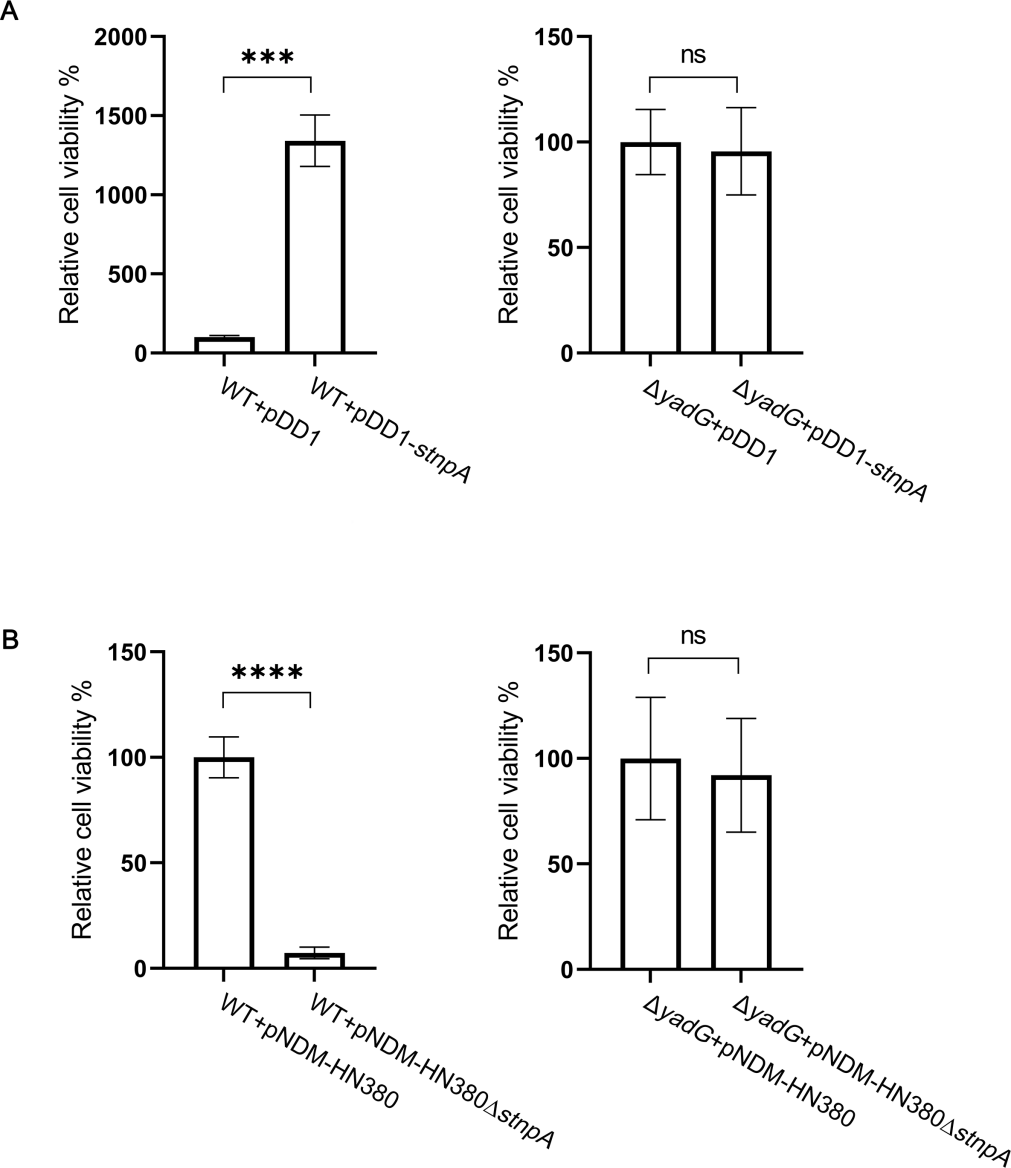
stnpA increases fosfomycin persistence in a YadG-dependent manner. (**A**) Analysis of fosfomycin persistence of strains overexpressing stnpA in the presence and absence of YadG. Persistence assay was conducted in J53/pDD1 (WT +pDD1), J53/pDD1-*stnpA* (WT +pDD1-*stnpA*), J53Δ*yadG*/pDD1 (Δ*yadG* + pDD1), and J53Δ*yadG*/pDD1-*stnpA* (Δ*yadG* + pDD1-*stnpA*). Data represented as means ± SD of three replicates. (**B**) Analysis of fosfomycin persistence of strains expressing endogenous stnpA in the presence and absence of YadG. Persistence assay was conducted in J53/pNDM-HN380 (WT +pNDM-HN380), J53/pNDM-HN380∆*stnpA* (WT +pNDM-HN380∆*stnpA*), J53Δ*yadG*/pNDM-HN380 (Δ*yadG* + pNDM-HN380), and J53Δ*yadG*/pNDM-HN380Δ*stnpA* (Δ*yadG* + pNDM-HN380∆ *stnpA*). Asterisks indicate significant differences between sample pairs specified by brackets: ns *P* > 0.05, *P < 0.05, **P < 0.01, ***P < 0.001, and ****P ≤ 0.0001 (two-tailed Student’s *t*-test).

To investigate if YadG is essential for the function of stnpA in controlling fosfomycin persistence, the null mutant of *yadG* in *E. coli* J53 (J53ΔyadG) was generated to study the persister formation under fosfomycin treatment. As shown in Fig. 5A, the cells overexpressing stnpA RNA dramatically enhanced the fosfomycin persistence in wild-type (WT) bacteria and increased the cellular survival from the antibiotic treatment (more than a 13-fold increase). On the contrary, the enhancement of the persistence totally disappeared in the null mutant of *yadG* overexpressing stnpA RNA. To investigate the functional significance of stnpA RNA on the plasmid and its interaction with YadG, the fosfomycin persistence was measured and compared between the bacteria (WT vs Δ*yadG*) carrying the pNDM-HN380 plasmid with and without stnpA RNA (pNDM-HN380 vs pNDM-HN380Δ*stnpA*). As shown in Fig. 5B, the fosfomycin persistence was significantly alleviated in the wild-type bacteria carrying pNDM-HN380Δ*stnpA,* whereas no effect was observed in the null mutant of *yadG*. Together, these findings demonstrated the regulation of stnpA RNA in fosfomycin persistence in the YadG-dependent manner.

### Accumulation of fosfomycin regulated by stnpA RNA in the YadG-dependent manner

**Fig 6:**
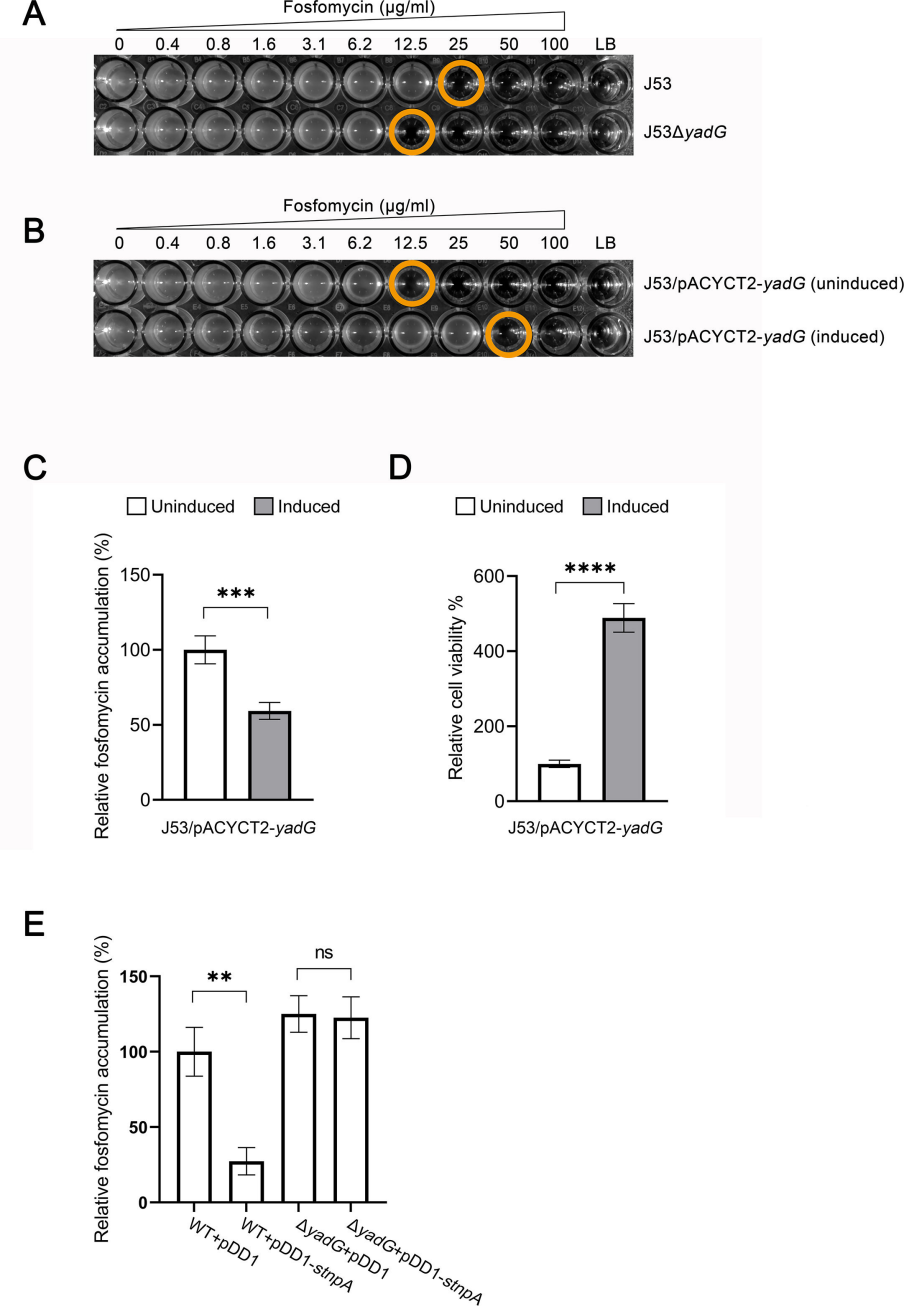
stnpA interacts with YadG to regulate fosfomycin accumulation. (**A**) Representative diagram of MIC determination against fosfomycin in J53 and J53Δ*yadG*. The final fosfomycin concentration ranging from 0 to 100 µg/mL. (**B**) Representative diagram of MIC determination against fosfomycin in J53/pACYCT2-*yadG* (IPTG-induced) and J53/pACYCT2-*yadG* (IPTG-uninduced). All tests were conducted in triplicate. The MIC value was expressed as the lowest fosfomycin concentration that inhibits bacterial growth. (**C**) Influence of YadG on intracellular accumulation of fosfomycin. Exponentially growing cultures of J53/pACYCT2-*yadG* cells were split into two groups: one was supplemented with 1 mM IPTG to induce the overexpression of YadG, while the other group remained uninduced as a control. After 2 hours of growth at 37°C, fosfomycin was added to the bacterial cultures to a final concentration of 2 mg/mL, followed by a 30-minute incubation at 37°C. Intracellular fosfomycin accumulation was quantified by LC-MS/MS. (**D**) Effect of YadG on fosfomycin persistence. Induced and uninduced J53/pACYCT2-*yadG* cells were treated with 100 µg/mL of fosfomycin for 2 hours. Cell viability was determined by counting the number of CFUs on LB agar plates using a serial dilution method. Percent cell viability represents the ratio of the subpopulation of persister cells after 2 hours of fosfomycin treatment over the total number of CFUs prior to fosfomycin addition, which is employed to evaluate the fosfomycin persistence of strains. (**E**) Quantification of fosfomycin accumulation in strains overexpressing stnpA in the presence and absence of YadG. Accumulation among these strains is given as the amount of fosfomycin (μg) in 10^6^ cells. WT +pDD1: J53/pDD1, WT +pDD1-*stnpA*: J53/pDD1-*stnpA*, Δ*yadG* + pDD1: J53Δ*yadG*/pDD1, and Δ*yadG* + pDD1-*stnpA*: J53Δ*yadG*/pDD1-*stnpA*. Data plotted are the mean of three independent tests. Statistically significant differences are indicated with asterisks (ns, not significant, *P  <  0.05, **P  <  0.01, ***P < 0.001, and ****P ≤ 0.0001).

We have shown that stnpA RNA enhanced the fosfomycin persistence of bacteria in a YadG-dependent manner. To explore the functional role of YadG on fosfomycin susceptibility, MIC measurements were conducted in various experimental strains including J53, J53Δ*yadG*, and J53/pACYCT2-*yadG* (induced and uninduced) strains. As shown in Fig. 6A, the presence of YadG in the parental strain (J53) showed twofold increase in the MIC compared with the null strain of YadG (J53Δ*yadG*). Interestingly, overexpression of YadG induced by IPTG in the J53/pACYCT2-*yadG* strain exhibited more enhancement of MIC to fosfomycin (Fig. 6B). These results suggest that YadG plays a role in the fosfomycin resistance of bacteria. It has been demonstrated that the control of antibiotic accumulation by membrane transporters is one of the hallmarks of bacterial antibiotic resistance and tolerance (35, 62). Since YadG is a hypothetical ATP-binding protein of an ABC transporter with predicted functions in antibiotic export (63, 64), we performed the antibiotic accumulation assay to determine the function of YadG in the export of fosfomycin. As shown in Fig. 6C and D, overexpression of YadG reduced the amount of fosfomycin in the cells and hence enhanced the cell viability, indicating the function of YadG in facilitating the transport of fosfomycin across the cell membrane.

Given that stnpA-controlled fosfomycin persistence is YadG-dependent and YadG is involved in the export of fosfomycin, we hypothesize that stnpA RNA regulates the accumulation of fosfomycin in the cells via the interaction of the YadG protein, leading to the persistence phenotype of the bacteria. As shown in Fig. 6E, overexpression of stnpA RNA reduced the accumulation of cellular fosfomycin compared with the controls. On the other hand, no significant difference was detected in the null mutant of YadG. Together with other results, the function of stnpA in the MDR plasmids was to control the fosfomycin persistence in a YadG-dependent manner by regulating the fosfomycin accumulation.

### stnpA is a possible regulator of YadG activity

**Fig 7:**
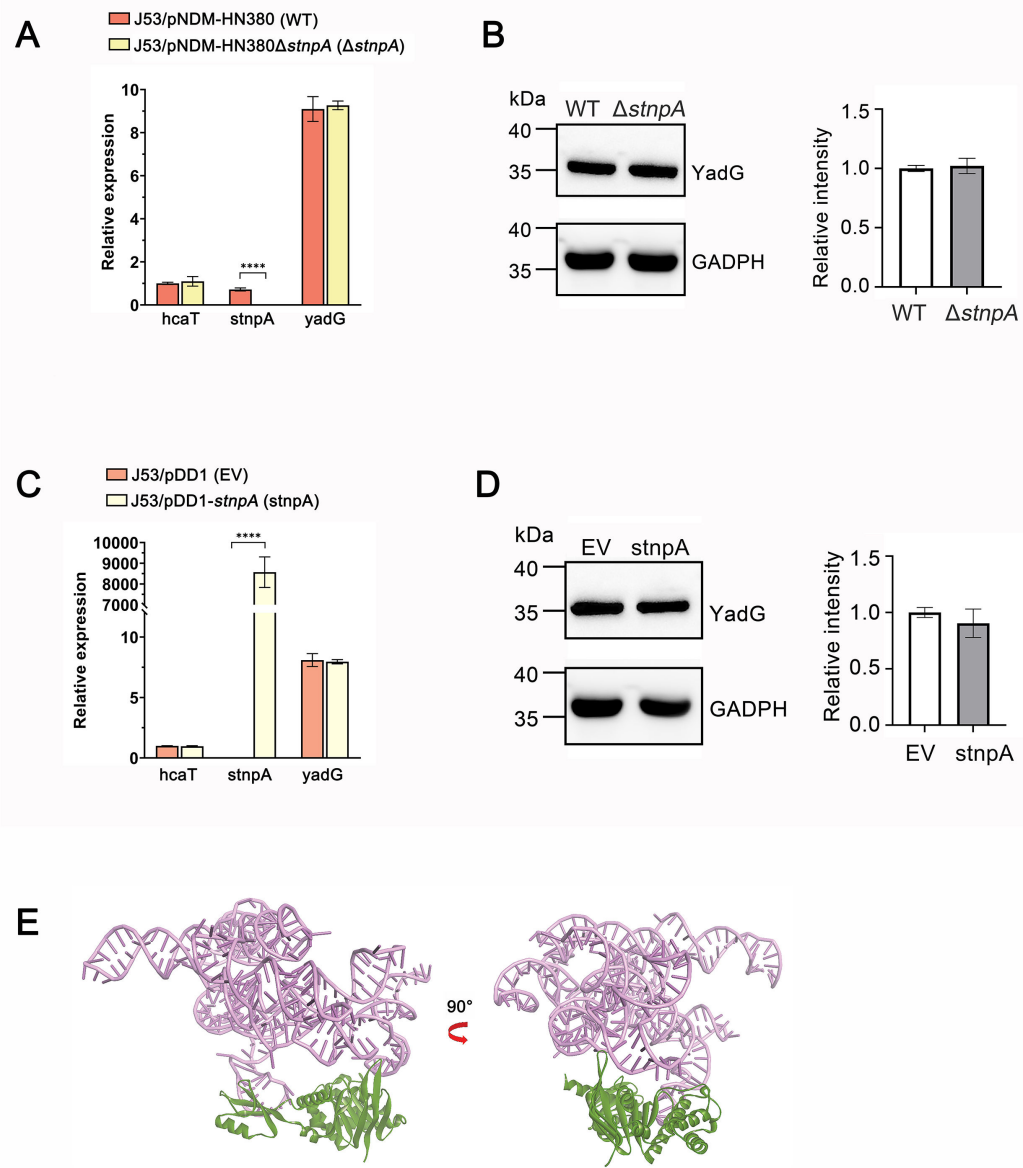
Effect of stnpA on mRNA and protein expression levels of YadG. (**A**) RT-qPCR was employed to quantify the mRNA expression level of yadG in J53/pNDM-HN380 (endogenous stnpA) and its control strain J53/pNDM-HN380Δ*stnpA*. Relative mRNA expression of selected genes was normalized to that of the housekeeping gene *gapA*, while the *hcaT* gene served as a reference to provide the assessment of the relative transcript abundance. (**B**) Western blot was conducted to assess YadG protein levels in J53/pNDM-HN380 in comparison to J53/pNDMHN380Δ*stnpA*. Left panel: representative Western blot showing the expression of YadG, with GAPDH serving as a loading control. Right panel: the relative intensity of YadG, which is defined as the intensity of the YadG protein normalized to GAPDH. (**C**) RT-qPCR analysis of yadG transcript levels in J53/pDD1-*stnpA* (overexpressing stnpA) and its control strain J53/pDD1. (**D**) Western blot analysis examined the expression of YadG protein in J53/pDD1-*stnpA* and J53/pDD1. Results represent means ± standard SD for three independent experiments. Statistical difference was determined at ****P ≤ 0.0001. (**E**) AlphaFold 3 model of stnpA–YadG interaction. stnpA is colored pink; YadG is colored green.

To investigate the regulatory role of stnpA in YadG expression, we compared both the transcription and protein expression levels of yadG in J53/pNDM-HN380 (with endogenous stnpA) versus J53/pNDM-HN380Δ*stnpA* (knockout of stnpA) and in J53/pDD1-*stnpA* (overexpressing stnpA) versus J53/pDD1 (empty vector control). As shown in Fig. 7A and C, the results of qPCR showed no significant differences in the transcription level of *yadG* in the presence and absence of stnpA. Noteworthily, the expression of stnpA in J53/pNDM-HN380 is similar to that of *hcaT*, a housekeeping gene often used for qPCR. Moreover, the same result was also observed in the YadG protein expression level, as indicated by Western blot analysis (Fig. 7B and D). These collective findings suggest that stnpA does not modulate the expression of YadG at either the transcriptional or translational level. Therefore, an alternative potential mechanism to enhance the function of YadG is the RNA–protein interaction between stnpA and YadG. To better understand the mechanism of YadG and stnpA binding, we employed AlphaFold 3 (65) to predict their structural interaction, as illustrated in Fig. 7E, and intriguingly, we observed two batches of extensive RNA–protein interaction between YadG and stnpA. The first one is the nucleotides 249–250 and 283–287 of stnpA with residues K12, Y14, P15, G16, V55, and N56 of YadG, whereas the other is the nucleotides 99–106 of stnpA with the residues I169, R173, P238, L283, S284, and R286 of YadG. These interactions suggest that the binding of stnpA may potentially induce the conformational change of YadG, which in turn enhances their activity to reduce the accumulation of fosfomycin.

## DISCUSSION

Transposons are segments of DNA that can shuffle to different positions in the genome of the cell, which are attributed to the changes of the genomic functions and play significant roles in the evolution of many genomes. However, little is known about the transposon-derived sRNAs and their functional roles in bacteria. Moreover, transposon-derived sRNAs are often found in the genome of bacteria but not on mobile elements such as plasmids. Recently, the newly found transposon-derived sRNAs were identified as the regulators of certain specific genes in various organisms, playing roles in bacterial adaptation against the challenges from the environments (18). In this study, we identified and characterized a novel small RNA (sRNA) called stnpA RNA, which locates within the gene of the Tn3 family transposon and is widely distributed on numerous prevalent drug-resistant plasmids in pathogenic bacteria. Moreover, this sRNA can interact with the putative ABC transporter ATP-binding protein YadG with high specificity and affinity and regulate the fosfomycin persistence of bacteria by reducing the effective concentration of fosfomycin in the cells. We believe that the combinatorial effect of the high mobility of the plasmids and effective transmission of transposons attribute to the rapid dissemination of the transposon-derived sRNA across a wide variety of species and provide fitness and survival advantages to the bacterial hosts. Noteworthy, our study also provided the first evidence of the function of the YadG protein as the ABC transporter membrane protein to facilitate the efflux of fosfomycin.

Antibiotic resistance and persistence were reported to be responsible for more than 700,000 global deaths yearly (66). Distinct from the resistant bacteria, the persistent ones remain viable but do not expand in the presence of antibiotics. Because of their ability to survive high antibiotic concentrations, which renders their eradication nearly impossible, and once the antibiotics are removed, the persisters resume growth (33), the presence of persister cells is a major cause of recalcitrance and relapse of persistent bacterial infections. Worse still, persisters are also shown to serve as reservoirs from which resistant mutants can emerge, which can eventually lead to complete antibiotic treatment failure (67, 68). Over the past decade, many environmental factors have been discovered to increase persister levels in bacteria, such as antibiotic exposure, nutrient limitation, and oxidative stress (33, 69, 70). Analysis of the transcriptome from samples enriched in persisters showed that numerous stress response genes were upregulated (71). Our study demonstrated, for the first time, that the stress of fosfomycin to the bacteria enhanced the expression of the plasmid-encoded sRNA, which interacted directly with YadG protein to expel the fosfomycin from the cell to reduce the intracellular effective concentration, leading to greater fosfomycin persistence and for bacterial survival.

The identification of stnpA and its functional role in fosfomycin persistence opens a new avenue for antimicrobial therapy. Targeting stnpA or stnpA-regulated processes holds promise for developing novel therapeutic strategies to disrupt persister cell formation and enhances the effectiveness of existing antibiotics to combat antibiotic resistance more effectively. Future studies of stnpA–YadG interaction could further investigate the mechanism of the fosfomycin transport and stnpA-mediated tolerance across different bacterial species. Such insights will be crucial for advancing our understanding of bacterial adaptation to antibiotic stress and for developing targeted interventions to combat persistent infections.

## SUPPLEMENTAL MATERIAL

Supplemental Material mbio.03814-24-s0001.docx Supplemental tables and figure.

Graphical Abstract mbio.03814-24-s0002.tif Visual abstract.

ASM does not own the copyrights to Supplemental Material that may be linked to, or accessed through, an article. The authors have granted ASM a non-exclusive, world-wide license to publish the Supplemental Material files. Please contact the corresponding author directly for reuse.
