# Supplementary material for: A novel plasmid-encoded transposon-derived small RNA reveals the mechanism of sRNA-regulated bacterial persistence
Source: mBio. 2025 Feb 25;16(4):e03814-24. doi: 10.1128/mbio.03814-24 (PMC11980398; doi:10.1128/mbio.03814-24)
Supplement: Supplemental Material — Supplemental tables and figure. [file mbio.03814-24-s0001.docx]

*Supplementary information*

**A novel plasmid-encoded transposon-derived small RNA reveals the mechanism of sRNA-regulated bacterial persistence**

Shu-Ling LIN^1,2^, Qi-Chang Nie^1,2^, Oi-Kwan Law^1,2^, Hoa-Quynh Pham^1,2^, Ho-Fai Chau^3^, Terrence Chi-Kong Lau^1,2^*

^1^Department of Biomedical Sciences, College of Veterinary Medicine and Life Science, City University of Hong Kong, Hong Kong SAR, China

^2^Tung Biomedical Sciences Centre, City University of Hong Kong, Hong Kong SAR, China

^3^Department of Applied Biology and Chemical Technology, The Hong Kong Polytechnic University, Hong Kong SAR, China

*Correspondence:

chiklau@cityu.edu.hk

Department of Biomedical Sciences, College of Veterinary Medicine and Life Science, City University of Hong Kong, Hong Kong SAR, China**Table S1**. Bacterial strains and plasmids used in the study.

| Strains and plasmids | Relevant characteristics | Reference or Source |
| --- | --- | --- |
| Strains | | |
| DH5α | F ^−^ φ80*lacZΔM15 Δ*(*lacZYA-argF*)*U169 recA1 endA1*  *hsdR17*(*rk- mk+*)*phoA supE44 λ-thi-1 gyrA96 relA1* | Invitrogen |
| BL21(DE3) | F ^−^ *ompT hsdSB(r_B_- m_B_-*) *gal dcm* (DE3) | Invitrogen |
| J53 | F ^−^ met pro Azi^R^ | (1) |
| J53Δ*yadG* | J53 with deletion of *yadG*, Azi^R^ | This study |
| CFT073 | UPEC isolate | (2) |
| HP16 | An DH5α derivative strain for B3H assay, Kan^R^ | This study |
| Plasmids | | |
| pNDM-HN380 | IncX3 plasmid carrying *stnpA* under control of its native promoter, CTX^R^ Amp^R^ | (3) |
| pSIJ8 | λ Red recombinase and flippase recombinase expression plasmid, Cam^R^ | (4) |
| pKD4 | PCR template for λ Red recombination system, Kan^R^ | (5) |
| pNDM-HN380Δ*tnpA* | pNDM-HN380 with deletion of *tnpA*, CTX^R^ Amp^R^ | This study |
| pNDM-HN380Δ*stnpA* | pNDM-HN380 with deletion of *stnpA*, CTX^R^ Amp^R^ | This study |
| pQH5 | Plasmid carrying a promoterless luxCDABE reporter gene cluster, Cam^R^ | This study |
| pQH5-*PstnpA* | 290 bp upstream region of *stnpA* (*PstnpA*) was cloned ahead of the promoterless luxCDABE reporter gene cluster in pQH5, Cam^R^ | This study |
| pQH5-*PstnpA* *SMu-1* | pQH5-*PstnpA* with SMu-1 mutation, Cam^R^ | This study |
| pQH5-*PstnpA* *SMu-2* | pQH5-*PstnpA* with SMu-2 mutation, Cam^R^ | This study |
| pQH5-*PstnpA* *SMu-3* | pQH5-*PstnpA* with SMu-3 mutation, Cam^R^ | This study |
| pQH5-*PstnpA* *SMu-4* | pQH5-*PstnpA* with SMu-4 mutation, Cam^R^ | This study |
| pQH5-*PstnpA* *SMu-5* | pQH5-*PstnpA* with SMu-5 mutation, Cam^R^ | This study |
| pQH5-*PstnpA* *SMu-6* | pQH5-*PstnpA* with SMu-6 mutation, Cam^R^ | This study |
| pDD1 | pT1 (6) deriviate plasmid with tac promoter (Ptac) and T7 terminator, Amp^R^ | This study |
| pDD1-*stnpA* | pDD1 carrying *stnpA* under the control of Ptac, Amp^R^ | This study |
| pET28a | Plasmid for overexpressing recombinant protein with an N-terminal His tag and/or a C-terminal His tag, Kan^R^ | Lab stock |
| pET28a-*yadG* | pET28a carrying *yadG* under the control of T7 promoter | This study |
| pBRα | pPrey vector used in B3H assay, encoding the α subunit of RNA polymerase, Amp^R^ | (7) |
| pBRα-*yadG* | pBRα encoding *yadG* tethered to the α subunit of RNA polymerase, Amp^R^ | This study |
| pCH1 | pBait vector used in B3H assay, encoding a single MS2 hairpin sequence, Str^R^ Spc^R^ | (8) |
| pCH1-*stnpA* | pCH1 encoding a hybrid RNA containing an MS2 hairpin upstream of *stnpA*, Str^R^ Spc^R^ | This study |
| pCH1-*antisense stnpA* | pCH1 encoding a hybrid RNA containing an MS2 hairpin upstream of *antisense stnpA*, Str^R^ Spc^R^ | This study |
| p35u4 | pAdapter vector used in B3H assay, encoding the a fusion protein consisting of the DNA binding-protein λCI and the RNA-binding coat protein from bacteriophage MS2, Cam^R^ | (9) |
| pACYCT2 | Cam^R^ | (10) |
| pACYCT2-*yadG* | pACYCT2 carrying *yadG* under the control of tac promoter, Cam^R^ | This study |

**Table S2**. Oligonucleotides used in the study.

| Name | Sequence (5′ -3′ ) | Description |
| --- | --- | --- |
| Δ*tnpA*-F | ACTTTGTCGTTTTTGGACGGAAAATCCCTAGAACCCCTCAGTGGGACGAAGCGATTGTGTAGGCTGGAGCTGCTTC | To delete *tnpA* from pNDM-HN380 |
| Δ*tnpA*-R | TTCTTGCATCGGTAACAGGGGTCTGACGCTCAGTGGAACGAAAACTCACGGCTGACATGGGAATTAGCCATGGTCC |  |
| Δ*stnpA*-F | TTGAAGTGGGGCGCATCAACAAAACGCTGTATCTGCTTAATTATATTGATGCGATTGTGTAGGCTGGAGCTGCTTC | To delete *stnpA* from pNDM-HN380 |
| Δ*stnpA*-R | AACGAAAACTCACGTTAAGCAACGTTTTCTGCCTCTGACGCCTCTTTTAAGCTGACATGGGAATTAGCCATGGTCC |  |
| Δ*yadG*-F | ACGATTTAAACCACAGATAAAGTGTAAGAACGTAAGGTAAGTAAAAATTTGCGATTGTGTAGGCTGGAGCTGCTTC | To make a  deletion of the *yadG* from J53 and CFT073 |
| Δ*yadG*-R | ATGGATCTCTTTCGCCCAGATGCTTTTTAGCGCCACCCAGTAAAGATGCAGCTGACATGGGAATTAGCCATGGTCC |  |
| *stnpA*-qPCR-F | CGCAGGGTGAAACACTGAATG | qPCR primer detects stnpA |
| *stnpA*-qPCR-R | CTGCCAGCGTGAAGGAATAATG |  |
| 5′ RACE adaptor | GCUGAUGGCGAUGAAUGAACACUGCGUUUGCUGGCUUUGAUGAAA | 5′ RACE RNA adaptor |
| 5′ RACE outer-F | GCTGATGGCGATGAATGAACACTG | To amplify first-round 5′ RACE PCR product |
| 5′ RACE outer-R | CAGTGTGCCCAGTTGATCTTCC |  |
| 5′ RACE inner-F | CGCGGATCCGAACACTGCGTTTGCTGGCTTTGATG | To amplify second-round 5′ RACE PCR product |
| 5′ RACE inner-R | TTTGACCGTGACAGATGGC |  |
| 3′ RACE adapter | GCGAGCACAGAATTAATACGACTCACTATAGGT12VN | 3′ RACE adapter |
| 3′ RACE outer-F | AACGCCGTCGTGTTATGGAA | To amplify first-round 3′ RACE PCR product |
| 3′ RACE outer-R | GCGAGCACAGAATTAATACGACT |  |
| 3′ RACE inner-F | CGCAGGGTGAAACACTGAATGA | To amplify second-round 3′ RACE PCR product |
| 3′ RACE inner-R | CGCGGATCCGAATTAATACGACTCACTATAGG |  |
| *PstnpA*-F | GGGGTACCAATCTTACTCAAACAGAGCCCA | To amplify stnpA promoter region from pNDM-HN380,  which is used to construct  pQH5-*PstnpA* |
| *PstnpA*-R | CGGAATTCATCAATATAATTAAGCAGATACAGCG |  |
| SMu-1-R | CGGAATTCATAAATATAATTAAGCAGATACAGCG | To generate mutations in stnpA promoter region by  site-directed mutagenesis |
| SMu-2-R | CGGAATTCATCAATATCATTAAGCAGATACAGCG |  |
| SMu-3-R | CGGAATTCATCAATATAATTAAGCATATACAGCGTTTTG |  |
| SMu-4-R | CGGAATTCATCAATATAATTAAGCAGATACAGCTTTTTGTTGATGCGCCCCAC |  |
| SMu-5-R | CGGAATTCATCAATATAATTAAGCAGATACAGCGTTTTGTGGATGCGCCCCAC |  |
| SMu-6-R | CGGAATTCATCAATATAATTAAGCAGATACAGCGTTTTGTTGATGCTCCCCACTTCAATG |  |
| pDD1-F | CTAGCATAACCCCTTGGGGCCTCTAAACGGGTCTTGAGGGGTTTTTTGAAGCTTGGCGTAATCATGGTC | To amplify pT1 backbone for pDD1 construction |
| pDD1-R | GGATCCTCTAGAGTCGACCCACACATTATACGAGCCGATGATTAATTGTCAAGGTACCGAGCTCGAATTCACTG |  |
| pDD1-*stnpA*-F | GGGGTACCTTGACAATTAATCATCGGCTCGTATAATGTGTGGGTCGACGATGAAGATTACCGCCGG | To amplify *stnpA* from pNDM-HN380 for cloning into pT1 to construct  pDD1-*stnpA* |
| pDD1-*stnpA*-R | CGGGATCCCAAAAAACCCCTCAAGACCCGTTTAGAGGCCCCAAGGGGTTATGCTAGTGGTCTCAGATGTCCTTTGGTC |  |
| stnpA-*in vitro*-F | GGGGTACCTAATACGACTCACTATAGGGGATGAAGATTACCGCCGG | To amplify the DNA template for *in vitro*  transcription of stnpA for RNA pull-down assay |
| stnpA-*in vitro*-R | CGGGATCCTGGTCTCAGATGTCCTTTGGTC |  |
| antisense stnpA-*in vitro*-F | GGGGTACCTAATACGACTCACTATAGGGTGGTCTCAGATGTCCTTTGGTC | To amplify the DNA template for *in vitro*  transcription of antisense stnpA for RNA pull-down assay |
| antisense stnpA-*in vitro*-R | CGGGATCCGATGAAGATTACCGCCGG |  |
| pET28a-*yadG*-F | GGAATTCCATATGATGACCATTGCACTGGAACTTCA | To amplify *yadG* from J53 to construct  pET28a-*yadG* |
| pET28a-*yadG*-R | CCCAAGCTTTCATGTGCGATCTCCTTGTTTTTCAT |  |
| *stnpA* with SP6-*in vitro*-R | ATTTAGGTGACACTATAGAATATGGTCTCAGATGTCCTTTGGTC | To amplify the DNA template for *in vitro*  transcription of stnpA for EMSA |
| SP6-FAM | FAM-ATTTAGGTGACACTATAGAATA | 5′ fluorescein (FAM)-labeled SP6 DNA primer |
| pBRα-*yadG*-F | TGGCTGAACAACTGGAAGCTGCAGCAGCAATGACCATTGCACTGGAACTTCA | To amplify *yadG* from J53 to construct  pBRα-*yadG* |
| pBRα-*yadG*-R | TGCGTCCGGCGTAGAGGATCCTCATGTGCGATCTCCTTGTTTTT |  |
| pCH1-*stnpA*-F | GACCTGCAGGCATGCAAGCTTGATGAAGATTACCGCCGGC | To amplify *stnpA* from pNDM-HN380 to construct  pCH1-*stnpA* |
| pCH1-*stnpA*-R | TCCGCCAAAACAGCCAAGCTTTGGTCTCAGATGTCCTTTGGTCA |  |
| pCH1-*antisense stnpA*-F | GACCTGCAGGCATGCAAGCTTTGGTCTCAGATGTCCTTTGGTCA | To amplify *antisense stnpA* from pNDM-HN380 to construct  pCH1-*antisense stnpA* |
| pCH1-*antisense stnpA*-R | TCCGCCAAAACAGCCAAGCTTGATGAAGATTACCGCCGGC |  |
| pACYCT2-*yadG*-F | GGAATTCCATATGAAAGAGGAGAAATTAACTATGAGAGGATCGCATCACCATCACCATCACGGTACCATGACCATTGCACTGGAAC | To amplify *yadG* from J53 to construct  pACYCT2-*yadG* |
| pACYCT2-*yadG*-R | CCTTAATTAATCATGTGCGATCTCCTTGTTTTTC |  |

**Table S3**. Proteins binding with stnpA (identified by Mass spectrometry).

| Description | Accession | Coverage |
| --- | --- | --- |
| Acetyl-coenzyme A carboxylase carboxyl transferase subunit beta OS=Escherichia coli O6:H1 (strain CFT073 / ATCC 700928 / UPEC) GN=*accD* PE=3 SV=2 | Q8FFH5 | 23.03 |
| ABC transporter ATP-binding protein *yadG* OS=Escherichia coli O6:H1 (strain CFT073 / ATCC 700928 / UPEC) GN=*yadG* PE=4 SV=1 | A0A0H2V455 | 22.73 |
| Iso_dh domain-containing protein OS=Escherichia coli O6:H1 (strain CFT073 / ATCC 700928 / UPEC) GN=*c2207* PE=4 SV=1 | A0A0H2V7X9 | 21.43 |
| Biotin carboxyl carrier protein of acetyl-CoA carboxylase OS=Escherichia coli O6:H1 (strain CFT073 / ATCC 700928 / UPEC) GN=*accB* PE=3 SV=1 | P0ABD9 | 14.96 |
| Sugar fermentation stimulation protein B OS=Escherichia coli O6:H1 (strain CFT073 / ATCC 700928 / UPEC) GN=*sfsB* PE=3 SV=1 | P0ACH2 | 10.87 |
| Putative ferredoxin-like protein yfhL OS=Escherichia coli O6:H1 (strain CFT073 / ATCC 700928 / UPEC) GN=*yfhL* PE=4 SV=1 | A0A0H2V9S8 | 8.14 |
| Putative tail fiber protein of prophage OS=Escherichia coli O6:H1 (strain CFT073 / ATCC 700928 / UPEC) GN=*c0977* PE=4 SV=1 | A0A0H2V5P0 | 8.08 |
| Murein DD-endopeptidase MepS/Murein LD-carboxypeptidase OS=Escherichia coli O6:H1 (strain CFT073 / ATCC 700928 / UPEC) GN=*mepS* PE=3 SV=1 | P0AFV5 | 5.32 |
| Phosphoribosylformylglycinamidine synthase OS=Escherichia coli O6:H1 (strain CFT073 / ATCC 700928 / UPEC) GN=*purL* PE=3 SV=3 | P0AFR5 | 4.37 |
| HTH araC/xylS-type domain-containing protein OS=Escherichia coli O6:H1 (strain CFT073 / ATCC 700928 / UPEC) GN=*c1810* PE=4 SV=1 | A0A0H2V761 | 3.69 |
| Oligopeptide transport system permease protein oppC OS=Escherichia coli O6:H1 (strain CFT073 / ATCC 700928 / UPEC) GN=*oppC* PE=3 SV=1 | A0A0H2V993 | 3.64 |
| Hypothetical ABC transporter ATP-binding protein yejF OS=Escherichia coli O6:H1 (strain CFT073 / ATCC 700928 / UPEC) GN=*yejF* PE=4 SV=1 | A0A0H2V901 | 2.84 |
| Glutamate--tRNA ligase OS=Escherichia coli O6:H1 (strain CFT073 / ATCC 700928 / UPEC) GN=*gltX* PE=3 SV=1 | Q8FFC9 | 2.76 |
| GABA permease OS=Escherichia coli O6:H1 (strain CFT073 / ATCC 700928 / UPEC) GN=*gabP* PE=4 SV=1 | A0A0H2VC98 | 2.67 |
| Rhamnulokinase OS=Escherichia coli O6:H1 (strain CFT073 / ATCC 700928 / UPEC) GN=*rhaB* PE=3 SV=1 | Q8FBD9 | 2.66 |
| Uncharacterized protein OS=Escherichia coli O6:H1 (strain CFT073 / ATCC 700928 / UPEC) GN=*c2063* PE=4 SV=1 | A0A0H2V7U7 | 2.42 |
| PAPS_reduct domain-containing protein OS=Escherichia coli O6:H1 (strain CFT073 / ATCC 700928 / UPEC) GN=*c0690* PE=4 SV=1 | A0A0H2V775 | 2.22 |
| Hypothetical σ-54-dependent transcriptional regulator ygeV OS=Escherichia coli O6:H1 (strain CFT073 / ATCC 700928 / UPEC) GN=*ygeV* PE=4 SV=1 | A0A0H2VA80 | 2.20 |
| Uncharacterized protein OS=Escherichia coli O6:H1 (strain CFT073 / ATCC 700928 / UPEC) GN=*c4898* PE=1 SV=1 | A0A0H2VD83 | 1.93 |
| Glycerol metabolism operon Regulatory protein OS=Escherichia coli O6:H1 (strain CFT073 / ATCC 700928 / UPEC) GN=*c1659* PE=4 SV=1 | A0A0H2V6Y2 | 1.87 |
| Adenine deaminase OS=Escherichia coli O6:H1 (strain CFT073 / ATCC 700928 / UPEC) GN=*ade* PE=3 SV=1 | Q8FBX7 | 1.87 |
| Uncharacterized protein OS=Escherichia coli O6:H1 (strain CFT073 / ATCC 700928 / UPEC) GN=*ydcM* PE=3 SV=1 | A0A0H2VAY9 | 1.50 |
| Phenylalanine--tRNA ligase beta subunit OS=Escherichia coli O6:H1 (strain CFT073 / ATCC 700928 / UPEC) GN=*pheT* PE=3 SV=1 | P59664 | 1.26 |
| Multidrug resistance protein MdtF OS=Escherichia coli O6:H1 (strain CFT073 / ATCC 700928 / UPEC) GN=*mdtF* PE=3 SV=1 | Q8FCI8 | 1.16 |
| Phosphoribosylformylglycinamidine synthase OS=Escherichia coli O6:H1 (strain CFT073 / ATCC 700928 / UPEC) GN=*purL* PE=3 SV=3 | Q8FF26 | 0.85 |


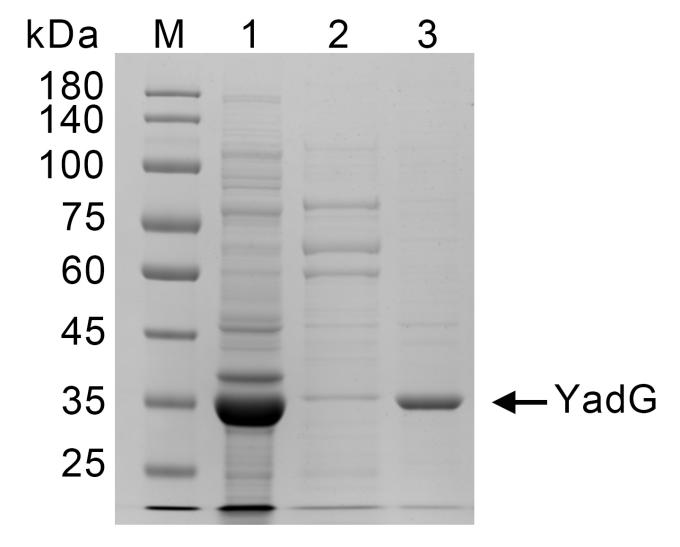


**Figure S1. Purification of** **His-tagged recombinant YadG.** M: molecular weight marker. The molecular mass standards are indicated on the left in kDa. Lane 1, bacteria lysate of BL21(DE3) cells overexpressing His-tagged recombinant YadG; lane 2, buffer exchanged eluate 1; lane 3, buffer exchanged eluate 2.

**References**

1. Yi, H., Cho, Y.-J., Yong, D. and Chun, J. (2012). Profiling bacterial community in upper respiratory tracts. BMC Infect. Dis. 14, 583.

2. Lloyd, A.L., Rasko, D.A. and Mobley, H.L. (2007) Defining genomic islands and uropathogen-specific genes in uropathogenic *Escherichia coli*. J. Bacteriol., 189, 3532-3546.

3. Ho, P.-L., Li, Z., Lo, W.-U., Cheung, Y.-Y., Lin, C.-H., Sham, P.-C., Chi-Chung Cheng, V., Ng, T.-K., Que, T.-L. and Chow, K.-H. (2012) Identification and characterization of a novel incompatibility group X3 plasmid carrying *bla* NDM-1 in Enterobacteriaceae isolates with epidemiological links to multiple geographical areas in China. Emerging Microbes Infect., 1, 1-6.

4. Jensen, S.I., Lennen, R.M., Herrgård, M.J. and Nielsen, A.T. (2015) Seven gene deletions in seven days: Fast generation of *Escherichia coli* strains tolerant to acetate and osmotic stress. Sci. Rep., 5, 1-10.

5. Datsenko, K.A. and Wanner, B.L. (2000) One-step inactivation of chromosomal genes in *Escherichia coli* K-12 using PCR products. PNAS, 97, 6640-6645.

6. Li, S.-K., Zhou, J.-W., Yim, A.K.-Y., Leung, A.K.-Y., Tsui, S.K.-W., Chan, T.-F. and Lau, T.C.-K. (2013) Organism-specific rRNA capture system for application in next-generation sequencing. PLoS One, 8, e74286.

7. Dove, S.L., Joung, J.K. and Hochschild, A. (1997) Activation of prokaryotic transcription through arbitrary protein-protein contacts. Nature, 386, 627-630.

8. Pandey, S., Gravel, C.M., Stockert, O.M., Wang, C.D., Hegner, C.L., LeBlanc, H. and Berry, K.E. (2020) Genetic identification of the functional surface for RNA binding by *Escherichia coli* ProQ. Nucleic Acids Res., 48, 4507-4520.

9. Wang, C.D., Mansky, R., LeBlanc, H., Gravel, C.M. and Berry, K.E. (2021) Optimization of a bacterial three-hybrid assay through in vivo titration of an RNA-DNA adapter protein. RNA, 27, 513-526.

10. Ponchon, L., Catala, M., Seijo, B., El Khouri, M., Dardel, F., Nonin-Lecomte, S. and Tisne, C. (2013) Co-expression of RNA-protein complexes in *Escherichia coli* and applications to RNA biology. Nucleic Acids Res., 41, e150-e150.
