# Supplementary figures and images for: A novel plasmid-encoded transposon-derived small RNA reveals the mechanism of sRNA-regulated bacterial persistence
Source: mBio. 2025 Feb 25;16(4):e03814-24. doi: 10.1128/mbio.03814-24 (PMC11980398; doi:10.1128/mbio.03814-24)

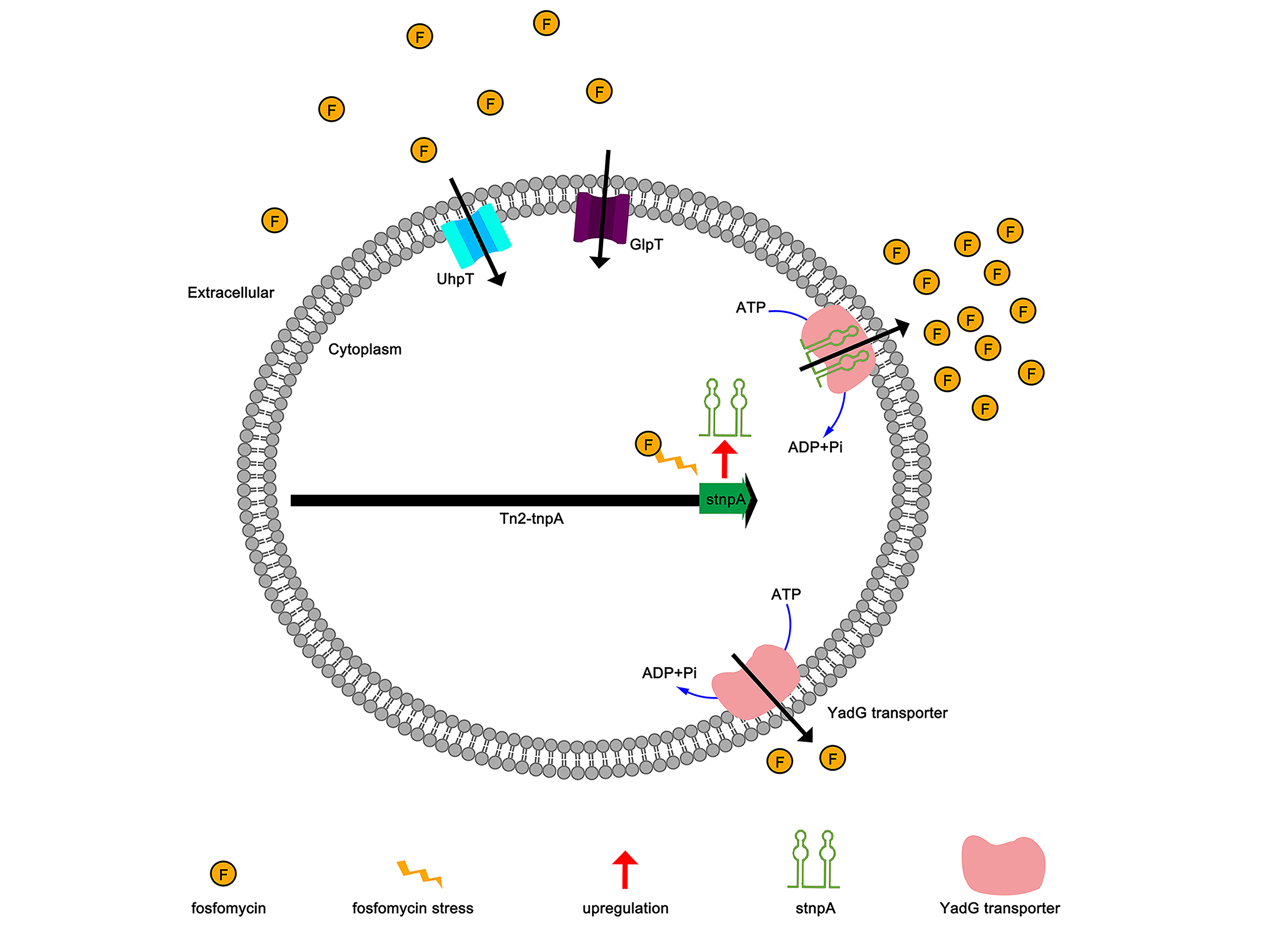

Supplement: Graphical Abstract — Visual abstract. [file mbio.03814-24-s0002.tif]
